# Assessment of Long-term Follow-up of Randomized Trial Participants by Linkage to Routinely Collected Data: A Scoping Review and Analysis

**DOI:** 10.1001/jamanetworkopen.2018.6019

**Published:** 2018-12-21

**Authors:** Tiffany Fitzpatrick, Laure Perrier, Sharara Shakik, Zoe Cairncross, Andrea C. Tricco, Lisa Lix, Merrick Zwarenstein, Laura Rosella, David Henry

**Affiliations:** 1 Ontario Strategy for Patient-Oriented Research (SPOR) SUPPORT Unit, Toronto, Ontario, Canada; 2 Dalla Lana School of Public Health, University of Toronto, Toronto, Ontario, Canada; 3 University of Toronto Libraries, Toronto, Ontario, Canada; 4 Li Ka Shing Knowledge Institute, St. Michael’s Hospital, Toronto, Ontario, Canada; 5 Institute of Health Policy, Management and Evaluation, University of Toronto, Toronto, Ontario, Canada; 6 Department of Community Health Sciences, University of Manitoba, Winnipeg, Manitoba, Canada; 7 Manitoba Centre for Health Policy, University of Manitoba, Winnipeg, Manitoba, Canada; 8 Department of Family Medicine, University of Western Ontario, London, Ontario, Canada; 9 Institute for Clinical Evaluative Sciences, Ontario, Canada; 10 Centre for Research in Evidence-Based Practice, Bond University, Gold Coast, Queensland, Australia

## Abstract

**Importance:**

Follow-up of participants in randomized trials may be limited by logistic and financial factors. Some important randomized trials have been extended well beyond their original follow-up period by linkage of individual participant information to routinely collected data held in administrative records and registries.

**Objective:**

To perform a scoping review of randomized clinical trials extended by record linkage to characterize this literature and explore any additional insights into treatment effectiveness provided by long-term follow-up using record linkage.

**Data Sources:**

A literature search in Embase, CINAHL, MEDLINE, and the Cochrane Register of Controlled Trials was performed for the period January 1, 1945, through November 25, 2016.

**Study Selection:**

Various combinations of search terms were used, as there is no accepted terminology. Determination of study eligibility and extraction of information about trial characteristics and outcomes, for both original and extended trial reports, were performed in duplicate.

**Data Extraction and Synthesis:**

Assessment of study eligibility and data extraction were performed independently by 2 reviewers. All analyses were descriptive.

**Main Outcomes and Measures:**

Outcomes in the pairs of original and extended trials were categorized according to whether any benefits or harms from interventions were sustained, were lost, or emerged during long-term follow-up.

**Results:**

A total of 113 extended trials were included in the study. Linkage to administrative and registry data extended follow-up by between 1 and 55 years. The most common interventions were pharmaceuticals (47 [41.6%]), surgery (19 [16.8%]), and disease screening (19 [16.8%]). End points most frequently studied through record linkage included mortality (88 [77.9%]), cancer (41 [36.3%]), and cardiovascular events (37 [32.7%]). One hundred four trial extensions (92.0%) were analyzed according to the original trial randomization. The reports provided details of 155 analyses of study outcomes. Seventy-four analyses (47.7%) identified statistically significant benefits in the trial extension phase. In 21 of these (28.4%), benefits were significant only in this period. Null results in both the original and extended trials were seen in 34 of the analyses (21.9%). Loss of significant benefits of an intervention were seen in 12 analyses (7.7%). Statistically significant harms were seen in 16 trial extension analyses (10.3%), and in 14 of these (87.5%), the harms were significant only in the trial extension phase.

**Conclusions and Relevance:**

Trial extension by linkage to routinely collected data is a versatile underused approach that may add critical insights beyond those of the original trial. Some beneficial and harmful outcomes of interventions are captured only in the extension phase of randomized trials.

## Key Points

**Question:**

Does follow-up of clinical trial participants by linkage to routinely collected data sources provide important insights into the long-term benefits and harms of treatment?

**Findings:**

This scoping review of the published literature found only 113 trials that had been extended by record linkage. Analysis showed that some benefits of treatment extend beyond the trial, and some harms of treatment only become apparent after the trial is complete.

**Meaning:**

The fate of patients after participation in clinical trials is a neglected topic, and the authors recommend that researchers routinely request permission from trial participants to study long-term treatment effects using linkage to routinely collected data.

## Teaser

This scoping review and analysis examines characteristics of randomized clinical trials that were extended by linkage to routinely collected data, including whether outcomes were sustained in the posttrial period.

## Introduction

Well-conducted randomized clinical trials remain the gold standard for generating estimates of efficacy, but follow-up times may be restricted by cost and logistical considerations. This reduces the capacity of trials to quantify long-term outcomes, including uncommon but serious harms of treatment.^1^ Trial extension by record linkage enables evaluation of long-term effectiveness of interventions, including end points that were not specified in the trial protocol.^2,3,4^ This has been aided by improved access to population-scale routinely collected health data sets, which can be linked to individual-level information held in other available databases long after the trials were terminated.

Recent examples of this approach include a 25-year follow-up of the Canadian National Breast Cancer Screening Study, achieved by linkage of trial participants to information held in cancer and vital statistics registries,^5^ and a 20-year follow-up of the West of Scotland Coronary Prevention study, achieved by linkage to administrative health data.^6^

There are several reasons for extending randomized trials. Most obvious is a desire to estimate the long-term benefits of an intervention.^7^ Extended follow-up may provide information on long-term harms, for instance the development of second malignancies after radiotherapy.^8^ Follow-up can enable study of the patterns and outcomes of treatment changes and co-interventions implemented after trial completion.

It is not clear how many trials have been extended by record linkage. This scoping review aimed to assess the frequency with which trial extension studies using routinely collected data have been performed, characterize the studies, and explore any additional insights they provided into treatment benefits and harms.

## Methods

The protocol for this review was registered in February 2017.^9^ In conducting the review, we followed published guidelines and the Preferred Reporting Items for Systematic Reviews and Meta-analyses (PRISMA) reporting guideline Extension for Scoping Reviews.^10,11,12^ The University of Toronto Health Sciences Research Ethics Board confirmed that review was not required as the study was limited to published information.

### Objectives of the Scoping Review

Our study aims raised several questions: (1) How many trials were extended by linkage to routinely collected data, and has the number increased over time? (2) In which countries were the studies performed? (3) What medical conditions and interventions were the targets of these initiatives? (4) Were data analyzed according to randomization? and (5) In what ways did the outcomes seen in the extended trials provide additional insights into the long-term benefits and harms of the interventions being studied?

### Literature Searching

We confined our search to the published literature in Embase, MEDLINE, CINAHL, and the Cochrane Register of Controlled Trials for the period January 1, 1945, to November 25, 2016^9^; details are provided in eTable 1 in the Supplement. The draft search strategy (for MEDLINE) was developed by 1 of us (L.P.). It was assessed by a second information scientist according to the Peer Review of Electronic Search Strategies (PRESS) checklist.^13^ Because this is an emerging area, there are no dedicated indexing terms, and there was considerable variability in how relevant studies were described. Our initial scan identified relevant studies that did not refer explicitly to data linkage in their abstracts. Accordingly, we searched for additional articles using the Related Articles feature in PubMed for articles included in the scoping review.^14^ The search strategy was limited to English-language articles, but there were no other restrictions. From the trial extension reports, we identified the reports of the original trials. All references were stored and shared using Reference Manager version 12.

### Study Eligibility and Data Extraction

Decisions about eligibility and data extraction (for the original trial and extension reports) were carried out by 2 independent reviewers. One investigator (T.F.) read all abstracts and full-text reports and extracted data from all eligible studies. The data extraction instrument was piloted by 2 investigators (T.F. and D.H.), with several modifications before use. To be eligible for full-text retrieval, trial extension reports had to describe follow-up of participants in a randomized clinical trial at least 1 year after completion of the trial using record linkage. To be eligible for data extraction, the study had to report linkage of trial participant information to routinely collected data sources (eg, vital statistics, disease registry, health administrative data). Studies that used a continuation of the normal trial follow-up processes or follow-up data obtained only from medical records at the home institution were excluded. Differences between reviewers were resolved by discussion. For some trials, there was more than 1 published extension report. In such cases, we used the longest follow-up study or, in cases where the longest follow-up study involved a subsample, we used the study that most closely matched the primary trial (in terms of study population, randomization, and the choice of end points). The information collected from the original and trial extension reports is provided in eTable 2 in the Supplement.

At all times, we used the end point definitions and analyses reported by authors and made no attempt to reclassify or recalculate the values published in the original or extended trial reports.

### Categorization of Trial Extension Outcomes

We considered several scenarios. A statistically significant advantage of an effective treatment might still be apparent years after trial completion despite uniform access to therapy, a so-called legacy effect. Conversely, the treatment benefits might decline over time, for instance in the case of waning immunity after vaccination. For trials showing apparent equivalence, or a statistically nonsignificant trend in favor of 1 treatment, similar patterns might be observed in the trial extension period, or an advantage of 1 treatment might emerge over time because of increased precision of the estimates of effectiveness. Similar patterns might also be observed for end points that were not specified in the original trial. For instance, the original trial might have measured the effectiveness of treatment on cancer recurrence, while the trial extension results reported on cancer-related mortality or all-cause mortality. Similar considerations can be applied to harms of treatment, which might continue, regress, or emerge during the trial extension period.

### Statistical Analysis 

Analyses were descriptive. We calculated percentages, medians, ranges, and interquartile ranges (IQRs). As this was a review of trials of a wide variety of interventions and end points there was no rationale for pooling data across studies and we did not test any hypotheses. In categorizing outcomes in the extended trial analyses as a significant benefit, a significant harm, a null result, or a loss of a significant benefit, we were guided by the statistical analyses reported by authors of the original and extended trials and did not reanalyze data. We did not assess the risk of bias of either the original trials or the trial extension studies. However, we did note whether posttrial interventions had been documented using routinely collected data and whether these had been included in trial analyses.

The main unit of analysis was the pairing of original and trial extension reports. Categorization of trial outcomes was complicated by the fact that some trial extension studies reported several analyses of different end points. We categorized extension study outcomes first by analyses and report here all permutations of persistence, loss or development of benefits, and harms that were seen across the pairs of trial reports. We then placed each pair of trial reports in a single category using a hierarchy that represented the findings of greatest clinical importance. We used this hierarchy to classify the studies (eTables 3 and 4 in the Supplement) and to provide clinical examples. The hierarchy was structured as follows: statistically significant benefits of treatment observed in the trial extension > statistically significant harms observed in the trial extension > null outcomes seen in the trial extension > benefits of the intervention lost during the trial extension period > outcome analysis was not according to randomization.

## Results

**Figure 1.**
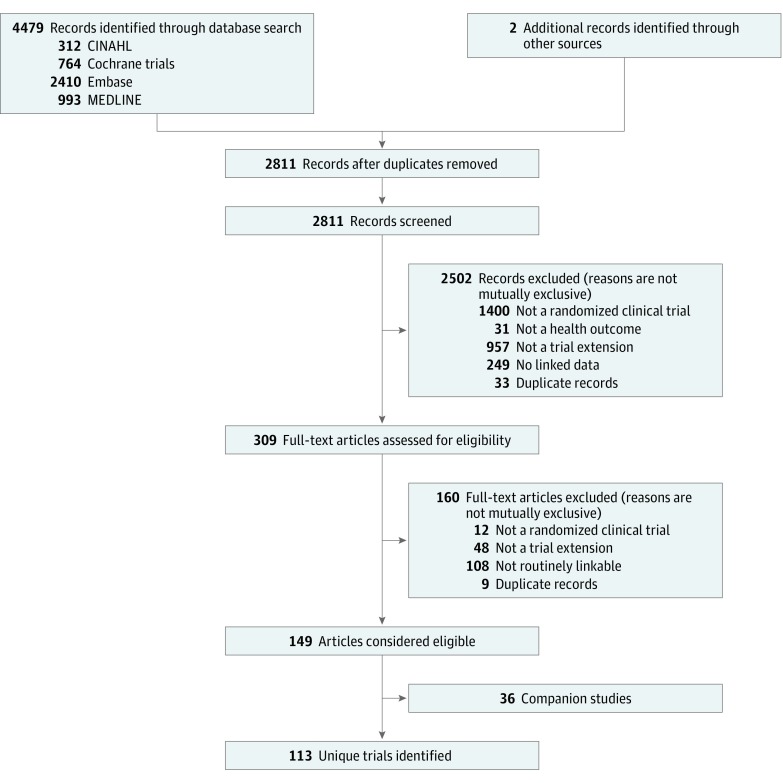
PRISMA Flow Diagram for Inclusion of Studies Into Trial Extension Scoping Review

From 2811 abstracts, we selected 309 full-text reports (Figure 1). One hundred sixty were excluded for the reasons summarized in Figure 1. Of the 149 remaining studies, 36 duplicate reports were excluded, yielding a total of 113 trial extension studies that met our inclusion criteria. Details of the references for the pairs of reports (original trial and extension reports) are provided in eTable 3 and eTable 4 in the Supplement.

### Original Trials

#### Countries of Conduct

Forty-nine trials (43.4%) were conducted in Nordic countries, 26 (23.0%) in the United States, and 25 (22.1%) in the United Kingdom. The other countries are listed in Table 1.

**Table 1.** Characteristics of the Original Trials Considered in the Scoping Review^a^

Study Characteristic	No. (%)
Countries of origin^b^	
Nordic	49 (43.4)
United States	26 (23.0)
United Kingdom	25 (22.1)
Netherlands	10 (8.8)
Australia or New Zealand	10 (8.8)
Europe (other)	9 (8.0)
Canada	8 (7.1)
Other	4 (3.5)
Intervention type	
Pharmaceutical	47 (41.6)
Surgery	19 (16.8)
Screening	19 (16.8)
Program (eg, general health, rehabilitation)	15 (13.3)
Diet	5 (4.4)
Psychological	5 (4.4)
Vaccine	4 (3.5)
Outcome type(s)^b^	
Mortality	67 (59.3)
Cardiovascular	36 (31.9)
Cancer	33 (29.2)
Renal or diabetes	10 (8.8)
Osteoporosis	6 (5.3)
Infectious diseases	6 (5.3)
Illicit drug use	5 (4.4)
Transplant	5 (4.4)
Pregnancy	4 (3.5)
Other (various)	45 (39.8)
Decade(s) conducted^b^	
2010s	2 (1.8)
2000s	35 (31.0)
1990s	74 (65.5)
1980s	42 (37.2)
1970s	11 (9.7)
1960s	1 (0.9)
1950s	3 (2.7)
1940s	1 (0.9)
1930s	1 (0.9)
Length of follow-up, y	
<1	24 (21.2)
1-4	51 (45.1)
5-9	32 (28.3)
10-19	5 (4.4)
20-29	1 (0.9)
Sample size, No. (%) [range]	
Quartile 1	28 (25) [68-462]
Quartile 2	28 (25) [462-1224]
Quartile 3	28 (25) [1224-6676]
Quartile 4	29 (26) [6676-291 523]
Used routinely collected data	
Yes	42 (37.2)
No	71 (62.8)
Industry funded	
Yes	49 (43.4)
No	49 (43.4)
Not stated	15 (13.3)

^a^ Some extension studies combined the participants from multiple original randomized clinical trials.

^b^ Categories are not mutually exclusive. Some multicenter trials were conducted in more than 1 country and spanned more than 1 decade.

#### Interventions and End Points

The most common interventions were pharmaceutical products (47 trials [41.6%]), surgery (including transplantation) (19 trials [16.8%]), and screening for disease (19 trials [16.8%]) (Table 1). The end points studied in the original trials were most commonly mortality (67 trials [59.3%]), cardiovascular disease events (blood pressure and lipid lowering treatments, and acute interventions for myocardial infarction) (36 trials [31.9%]), and cancer (33 trials [29.2%]). However, the range of interventions and end points was diverse (Table 1).

#### Trial Commencement Dates and Follow-up Periods

Most of the original trials took place in the 1980s and 1990s (Table 1). However, 6 (5.3%) commenced before 1970, the oldest being a randomized placebo-controlled trial of the BCG vaccine, which commenced in 1935.^15^ Follow-up periods of the original trials ranged from less than 1 year to more than 20 years (Table 1).

### Trial Extension Studies

**Figure 2.**
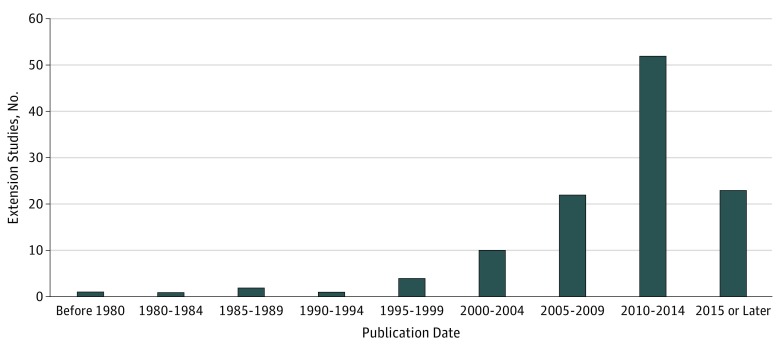
Numbers of Trial Extension Studies Published Over Time Of 113 total studies included in our review, 9 were published in the first 15 weeks of 2016.

The frequency of publication was highest in the most recent years of the study (Figure 2).

#### Extended Follow-up Times

The median (IQR) additional follow-up achieved by record linkage was 8 (4.5-13.8) years. The overall range of additional follow-up varied from 1 to 50 years. The total follow-up times (original plus trial extensions) ranged from 3 to 55 years, with a median (IQR) of 10.9 (7.3-19) years (Table 2).

**Table 2.** Characteristics of the Trial Extension Studies Included in the Scoping Review

Study Characteristic	No. (%)
Extension planned	
Yes	19 (16.8)
No	44 (38.9)
Not stated or unclear	50 (44.2)
Length of posttrial follow-up, y	
1-4	32 (28.3)
5-9	32 (28.3)
10-19	34 (30.1)
20-29	11 (9.7)
30-39	2 (1.8)
40-49	0
50-59	1 (0.9)
Total study follow-up, y	
1-4	6 (5.3)
5-9	34 (30.1)
10-19	48 (42.5)
20-29	21 (18.6)
30-39	4 (3.5)
40-49	0
50-59	1 (0.9)
Sample size, No. (%) [range]	
Quartile 1	28 (25) [47-322]
Quartile 2	28 (25) [322-1222]
Quartile 3	28 (25) [1222-5804]
Quartile 4	29 (26) [5804-427 010]
Analyzed according to original randomization	
Yes	104 (92.0)
No	9 (8.0)
Outcome type(s)^a^	
Mortality	88 (77.9)
Cancer	41 (36.3)
Cardiovascular	37 (32.7)
Transplant	6 (5.3)
Renal or diabetes	6 (5.3)
Osteoporosis	3 (2.7)
Other (various)	29 (25.7)
Authorship includes the original trial investigators	
Yes	105 (92.9)
No	6 (5.3)
Unclear	2 (1.8)
Industry funded	
Yes	25 (22.1)
No	66 (58.4)
Not stated	21 (18.6)

^a^ Categories are not mutually exclusive.

#### Sample Sizes

The original trial sample sizes varied from 68 to 291 523 with a median (IQR) of 1224 (460-6595). Sample sizes in the extension studies ranged from 47 to 247 010, with a median (IQR) of 1222 (322-5378).

#### Details of Analyses

Analyses were reported according to the original randomization in 104 of the trial extension reports (92.0%) (Table 2). In 86 of the 99 reports that provided information (86.9%), results were reported by intention to treat. Access to relevant intervention data after the trial concluded was reported in 18 studies (15.9%). This information was used in 7 studies (6.2%).

#### Clinical End Points Measured in the Original and Extended Trials

Mortality statistics (cause-specific mortality, all-cause mortality, or both) were documented in 88 trial extension studies (77.9%), compared with 67 of the original trials (59.3%) (Table 1 and Table 2). Cancer end points (progression, recurrence, and cancer-related mortality) were reported in 41 extended studies (36.3%), compared with 33 of the original trials (29.2%) (Table 1 and Table 2). Cardiovascular end points (cardiovascular events and deaths) were documented with similar frequencies in the original (36 [31.9%]) and extended (37 [32.7%]) trial reports.

#### Research Ethics Approvals

Research ethics reviews were reported for 44 of the extension studies (38.9%). In 39 (34.5%), it appeared that ethics review for the extension study had not been requested, and in 30 (26.5%) no judgment could be made.

#### Details of Data Linkage

In 36 studies (31.9%) linkage involved only vital statistics registries. Health administrative data documenting hospital discharges were used in 31 studies (27.4%), cancer registries in 28 (24.8%), and specialized registries in 13 (11.5%). Data linkage methods were described in 49 (43.4%) of the studies. Of these, 33 (67.3%) reported using deterministic methods and 16 (32.7%) reported probabilistic methods.

### Categorization According to Analyses of Study Outcomes

In total, the 113 trial extension reports provided details of 155 analyses of study outcomes. These are categorized in Table 3. Seventy-four analyses (47.7%) identified statistically significant benefits in the trial extension phase. In 21 of these (28.4%), benefits were significant only in this period. Null results in both the original and extended trials were seen in 34 of the analyses (21.9%). Loss of significant benefits of an intervention were seen in 12 analyses (7.7%). Statistically significant harms were seen in 16 analyses (10.3%), and in 14 of these (87.5%), the harms were significant only in the trial extension phase.

**Table 3.** Summary of Results of Trial Extension Studies^a^

Patterns of Outcomes Reported in the Trial Extension Study	No. (%)	Comments
Significant benefits of intervention seen in original and extended trial using the same end points as in the original trial	42 (27.1)	Using the same end point measures, but defined using administrative data in the extended trial
Significant benefits of intervention seen in original and extended trial with different end points in the extended trial	11 (7.1)	Different end points could be, for example, cardiovascular deaths rather than cardiovascular events
Significant benefits of intervention seen only in the extended trial using the same end points as in the original trial	6 (3.9)	Using the same end point measures, but defined using administrative data in the extended trial
Significant benefits of intervention seen only in the extended trial using different end points in the extended trial	15 (9.7)	
Equivalence of intervention seen in original and extended trial	20 (12.9)	Using the same end point measures, but defined using administrative data in the extended trial
Equivalence of intervention seen in original and extended trial with different end points in the extended trial	14 (9.0)	
Significant benefits of intervention seen in the original trial were no longer significant in the extended trial	12 (7.7)	Using the same end point measures, but defined using administrative data in the extended trial
Significant harms of intervention seen only in the extended trial	14 (9.0)	
Significant harms seen in original and extended trial	2 (1.7)	
Outcomes in the extended trial were not analyzed according to randomization	19 (12.3)	For example, observational study of treated cohort only

^a^ Includes a total of 155 analyses from 113 study reports.

### Categorization of Original and Extended Trial Reports

The 113 study pairs are categorized in eTable 3 and eTable 4 in the Supplement.

#### Studies That Found Long-term Benefits in the Trial Extension Phases

Sixty-one extension studies (53.9%) described a statistically significant long-term benefit of the original trial intervention. In 42 of these (68.9%) the benefit appeared to be a continuation what was seen in the original trial. Examples included a trial of thrombolysis in myocardial infarction documenting a reduction in all-cause mortality at 1 year that persisted after 10 years^16^; a trial of aggressive lowering of LDL cholesterol by statins that reduced the rate of revascularization by 30% after 4 years, with a similar reduction still apparent after 7.5 years^17^; and a 55-year follow-up of the first trial of BCG vaccination that found a persistent level of protection against tuberculosis.^15^ In 21 studies (18.6%), statistically significant long-term benefits were seen only in the trial extension phase. Four of these reported reductions in all-cause mortality,^18,19,20,21^ 4 reported reductions in cardiac events,^21,22,23,24^ and 2 reported reduced rates of cancer.^25,26^

#### Harms of Treatment in Trial Extension Phases

Thirteen of the trial extension reports (11.5%) described long-term harms of the interventions, including 4 reports of long-term harms in patients randomized to pelvic or chest radiotherapy for colorectal, endometrial, or breast cancer: venous and arterial thrombosis, adhesions, intestinal fistulae, and second cancers.^27,28,29^ There were 2 reports of possible long-term harms of estrogens: atrial fibrillation in postmenopausal women and benign lesions of the cervix in female offspring of women who received them in pregnancy.^30,31^ Two reports described possible harms of blood transfusion: reduced survival in trial participants who received buffy coat–depleted red cells and in recipients of autologous red cell transfusions.^32,33^

#### Trials With Null Results

Twenty-two of the trial extension reports (19.5%) found no significant difference in outcomes between intervention and control groups. Examples included the following: no increase in cardiotoxicity when epirubicin replaced methotrexate in chemotherapy for breast cancer^34^; a reduction in cardiac events, but not overall survival, in high-risk elderly patients treated with pravastatin^35^; a trial of BCG vaccine in patients with bladder cancer finding no improvement in overall survival compared with mitomycin C despite promising results in the original trial^36^; and a follow-up study of children with in utero exposure to progestogen that did not lead to long-term harms for child health and development.^37^

#### Loss of Intervention Benefits

Intervention benefits seen in the original trial were lost in the trial extension phase in 6 studies (5.3%). Examples included the following: after an early advantage of mycophenolate mofetil over azathioprine in graft rejection in the first 6 months after renal transplantation, the extension analysis found no differences in long-term patient or graft survival^38^; an early reduction in cardiac events was observed in patients who received early aggressive (rather than conservative) intervention for unstable angina, but long-term follow-up found no reduction in all-cause mortality and cardiovascular deaths^39^; and a significant reduction in breast cancer mortality in a mammography-screened group in the first 10 years after diagnosis, but not thereafter.^40^

## Discussion

Against a background of more than 20 000 randomized trials registered each year, this scoping review identified a small number of reports of trials that had been extended by linkage to registry and administrative data to evaluate long-term outcomes of trial interventions.^41^ Nordic countries were overrepresented in this literature, a testament to their commitment to conducting trials and the value placed on maintaining high-quality registries that enable data linkage.^42^

The versatility of trial extension by data linkage is illustrated here by the wide variety of findings. These include observations of a possible legacy effect of statin use^43^; a 55-year protective benefit of BCG vaccination^15^; evidence of cancer prevention with hepatitis B virus and human papillomavirus vaccines^44,45^; confirmation that in-utero exposure to progestogens does not lead to child health or developmental problems^37^; and quantification of an increased risk of second malignancies long after radiotherapy for breast or endometrial cancer.^29,46^ We highlight these studies to illustrate the value of trial extension using linkage to routinely collected data and do not claim that these study findings are definitive estimates of the benefits and harms of the interventions.

Because of the heterogeneity of topics, we did not perform meta-analyses of trial extension outcomes. But this has been done recently by Nayak and colleagues^47^ while exploring posttrial statin legacy effects on all-cause mortality. They suggest that most effect is seen in primary prevention studies.

Our data showed an increasing rate of publication of trial extension studies, albeit from a low base. Growing awareness of this approach and greater availability of linkable registry data may lead to more extension studies being planned as part of the original trials. Increasing access to randomized trial data through developments such as the AllTrials movement may encourage independent groups to perform participant data linkage.^48^ This provides an opportunity to both reproduce the original trial analyses and determine long-term outcomes, which was not done in any of the studies reviewed here.

The reports we retrieved did not document the costs of follow-up of trial participants; however, record linkage is inexpensive. The cost of the extension of the West of Scotland Coronary Prevention trial (N = 6595) was stated to be £15 000 ($19 600), a fraction of the cost of the original trial.^49^ In their recent review of long-term follow-up of large randomized clinical trials published between 2006 and 2017, Llewellyn-Bennett and colleagues^4^ found that costs varied from thousands of dollars using record-linkage to millions of dollars with clinical follow-up.

### Methodological and Reporting Issues

It was not clear how often trial extension had been planned as part of the original trial. This raises the possibility of post hoc selection of study end points and/or analyses, an important source of bias.^50^ In most of the extension studies, the end points were those used in the original trials, except that they were quantified using routinely collected data, such as vital statistics, cancer registries, and hospital discharge diagnoses. While posttrial analyses of linked data are observational, we believe they have clear advantages over traditional studies of long-term outcomes because the original exposure was determined by randomization. The initial randomization step minimizes selection bias, and most trials demonstrate good control at baseline for confounders, but the long follow-up periods provide many opportunities for treatment switches and co-interventions. Our data show that these are usually undocumented. This limits capacity to adjust for time-dependent variables, which can distort estimates of intervention effectiveness.

We did not assess the risk of bias in the trials included in the review, which would have been necessary if we were conducting meta-analyses. In their recent systematic review of posttrial follow-up methodology in large randomized clinical trials, Llewellyn-Bennett and colleagues^4^ found a generally low risk of bias and similar attrition rates with different follow-up methods. However, bias assessment appears to have been limited to the randomized phase of the trial and may not have taken account of time-varying confounding in the posttrial phase.

Some information was incompletely reported in the studies we reviewed, including whether the study was preplanned, the quality and accuracy of the data used, and the type and success of the linkage method. It was sometimes unclear whether ethics approval had been specifically sought for the extension study or whether specific funding sources had been secured for the long-term study.

It is beyond the scope of this article to provide opinions on all standards that should apply to this type of work, but we can identify several key topics. Important activities to be included in the original trial planning include (1) ethics approval for data linkage and analysis and inclusion of this procedure in the original consent forms and (2) awareness by institutional ethics committees of the need to preserve trial records, including information required for data linkage. The value of trial extension reports could be improved by adherence to reporting standards. The RECORD (Reporting of Studies Conducted Using Observational Routinely-Collected Data) collaborative provides guidance and a checklist developed from the original STROBE (Strengthening the Reporting of Observational Studies in Epidemiology) guidelines.^51^ In addition, there are plans to develop a CONSORT (Consolidated Standards of Reporting Trials) extension for randomized clinical trials using cohorts and routinely collected health data.^52^

### Accuracy of Routinely Collected Data

Administrative data are usually limited to major events that lead to hospitalization, death, and notifiable diseases. In such cases, the accuracy of routinely collected data is assumed to be high. It was notable that none of the studies we reviewed took the opportunity to calibrate the initial trial end point frequencies against contemporaneous administrative data before using the latter to evaluate long-term outcomes. Changing coding practices or inaccuracies and alterations in disease definitions can affect interpretation. Administrative records generally lack information on vital signs and laboratory and diagnostic test results.^53,54^ Some billing data for community care include diagnostic information (eg, in Ontario, Canada), but this appears uncommon.^55^ Data on prescribing or dispensing of medicines are usually available and are accurate, but access to linkable routine laboratory data is variable.^54^ Important clinical information, such as body weight, smoking history, and blood pressure are usually absent from administrative data.^56^ Thus, some trial extension work will be limited by lack of access to accurate clinical data with which to explain variations in trial outcomes.

### Limitations 

It is likely that our literature search missed studies. However, we think that those we selected are representative of the wider literature. Our difficulty with searching underscores the need for agreement on terminology to ensure consistent indexing of relevant studies. As noted in the preceding paragraphs, we did not reanalyze the aggregate data or assess the accuracy of the authors’ statistical analysis. We did not critically appraise the articles. In categorizing long-term outcomes, we used an arbitrary approach based on authors’ reported statistical analyses, and our categories probably overlap. However, our conclusions are broad, based on descriptive analyses, and unlikely to be sensitive to misclassification.

## Conclusions

Trial extension by linkage to routinely collected data is a versatile, underused approach that may add critical insights beyond those of the original trial. Some beneficial and harmful outcomes of interventions are captured only in the extension phase of randomized trials.

## Notes

Supplement. **eTable 1.** MEDLINE Search Strategy **eTable 2.** Summary of Data Abstraction Guide **eTable 3.** Complete List of References for the 113 Included Extension Studies and Their Corresponding Original Trial Reports, According to Outcome Categorization **eTable 4.** Details of Participants Interventions and Outcomes Measured in the Original and Extended Trials Included in the Review
