# Supplementary material for: Assessment of Long-term Follow-up of Randomized Trial Participants by Linkage to Routinely Collected Data: A Scoping Review and Analysis
Source: JAMA Netw Open. 2018 Dec 21;1(8):e186019. doi: 10.1001/jamanetworkopen.2018.6019 (PMC6324362; doi:10.1001/jamanetworkopen.2018.6019)
Supplement: Supplement. — eTable 1. MEDLINE Search Strategy eTable 2. Summary of Data Abstraction Guide eTable 3. Complete List of References for the 113 Included Extension Studies and Their Corresponding Original Trial Reports, According to Outcome Categorization eTable 4. Details of Participants Interventions and Outcomes Measured in the Original and Extended Trials Included in the Review [file jamanetwopen-1-e186019-s001.pdf]

Supplementary Online Content

Fitzpatrick T, Perrier L, Shakik S, et al. Assessment of long-term follow-up of randomized trial participants by linkage to routinely collected data: a scoping review and analysis. *JAMA Netw Open*. 2018;1(8):e186019. doi:10.1001/jamanetworkopen.2018.6019

eTable 1. MEDLINE Search Strategy

eTable 2. Summary of Data Abstraction Guide

eTable 3. Complete List of References for the 113 Included Extension Studies and Their Corresponding Original Trial Reports, According to Outcome Categorization

eTable 4. Details of Participants Interventions and Outcomes Measured in the Original and Extended Trials Included in the Review

This supplementary material has been provided by the authors to give readers additional information about their work.

© 2018 Fitzpatrick T et al. *JAMA Network Open.*

eTable 1. MEDLINE Search Strategy

Database: Ovid MEDLINE(R) <1946 to November Week 4 2016>,
Ovid MEDLINE(R) In-Process & Other Non-Indexed Citations <November 25, 2016>
Search Strategy:

1 (extension and trial).ti.
2 randomized trial.mp. [RCT filter - validated, highly sensitive]
3 randomized trial.pt.
4 random:.mp.
5 tu.xs.
6 or/2-5
7 ("2 year" adj "follow-up").tw.
8 ("2$ year" adj "follow-up").tw.
9 ("3 year" adj "follow-up").tw.
10 ("3$ year" adj "follow-up").tw.
11 ("4 year" adj "follow-up").tw.
12 ("4$ year" adj "follow-up").tw.
13 ("5 year" adj "follow-up").tw.
14 ("5$ year" adj "follow-up").tw.
15 ("6 year" adj "follow-up").tw.
16 ("6$ year" adj "follow-up").tw.
17 ("7 year" adj "follow-up").tw.
18 ("7$ year" adj "follow-up").tw.
19 ("8 year" adj "follow-up").tw.
20 ("8$ year" adj "follow-up").tw.
21 ("9 year" adj "follow-up").tw.
22 ("9$ year" adj "follow-up").tw.
23 ("1$ year" adj "follow-up").tw.
24 ("long term" adj "follow-up").tw.
25 *Follow-Up Studies/
26 *Longitudinal Studies/
27 (longitudinal adj stud$).tw.
28 (post adj trial?).tw.
29 (after adj trial?).tw.
30 (passive adj surveillance).tw.
31 or/7-30
32 Medical Records/
33 Health Records, Personal/
34 Medical Record Linkage/
35 Medical Records Systems, Computerized/
36 Electronic Health Records/
37 Health Information Exchange/
38 Nursing Records/
39 Records as Topic/
40 exp Hospital Information Systems/
41 exp Registries/
42 registry.tw.
43 registries.tw.
44 (data adj link$).tw.
45 (link$ adj2 data).tw.
46 (death adj certificate?).tw.
47 "vital stat$ data$".tw.
48 EHR.tw.
49 EMR.tw.
50 (medical adj record?).tw.
51 (health adj record?).tw.
52 (patient adj record?).tw.
53 (nursing adj record?).tw.
54 (national adj4 index).tw.
55 (national adj4 register?).tw.
56 (national adj4 data$).tw.
57 (administrative adj4 data$).tw.
58 (administrative adj4 record$).tw.
59 (population-based adj4 data$).tw.
60 or/32-59
61 6 and 31 and 60
62 1 or 61
63 exp Animals/ not (Humans/ and exp Animals/)
64 62 not 63
65 limit 64 to english language

© 2018 Fitzpatrick T et al. *JAMA Network Open.*

eTable 2. Summary of Data Abstraction Guide

Bibliometrics

- Original trial reference
- List of other companion trial references (if applicable)

Original Trial characteristics

- NCT Number (or other trial registration number, if registered in another country)
- Country(ies) of origin
- Intervention(s) applied, include cointerventions, if applicable.
- Intervention type
- Outcomes originally outlined (include primary and secondary, if applicable)
- Time frame; i.e., calendar dates for which the study was conducted
- Length of intervention (e.g. in months, years - as per original trial reported)
- Average follow-up time (in months), if provided.
- Baseline sample size
- Study inclusion criteria
- Did the original trial use routinely collected (administrative/ vital statistics/ registry) data to measure primary or secondary trial outcomes? If yes, specify.
- Main study findings (i.e., those reported in the abstract)
- Funders (if not stated, specify)
  - Did the original study had industry sponsorship?

Follow-up trial characteristics

- Was the extension planned as part of the original trial? (Yes/No/Can't tell)
- Was a clear reason given to perform extension?
  - If so, specify; e.g. to determine the long-term benefits of treatment?
- Additional follow-up (e.g. years, months) offered through trial extension.
- Average total follow-up (in months) - or maximum follow-up time when multiple trials were included or where mean/ median are not explicitly stated
- Did the follow-up involve subgroups of the original trial population? If so, briefly describe.
- Sample size used for follow-up study
- Were outcomes reported according to randomization?
- List the outcomes reported by the follow-up study. State primary and secondary outcomes.
- Do the outcomes reported in the follow-up study differ from those reported in the original trial report? Describe.
- Did the authors also report outcomes in an additional population? If yes, specify (e.g. individuals in a registry).
- Main results of follow-up study (i.e., those reported in the abstract)
- What additional information did the trial extension provide? E.g. original intervention effects were sustained/lost. Were there new findings involving other outcomes?
- Were any of the authors of the original study involved in the follow-up study?

© 2018 Fitzpatrick T et al. *JAMA Network Open.*

Follow-Study Data and Funding Sources<br>• State the types of routinely collected data used in the follow-up study, e.g. vital statistics, administrative claims data, registry data, etc.<br>• Did the authors use data other than vital statistics or population registries?<br>• Was the method of linkage used reported or referenced? If so, specify (e.g. probabilistic, deterministic)<br>• Do the authors report the % (or number) of study participants successfully linked? If so, record.<br>• Did the authors report if Research Ethics Board (REB) approval was sought?<br>o Please record any mention to ethics and page number.<br>o Was REB approval specific to the long-term follow-up study?<br>• Did the authors have access to details of interventions after the original trial was closed?<br>o If yes, specify.<br>• If they had access to trial intervention data, how did they use it?<br>• How did the authors state how they treated the analysis? i.e., per-protocol, intention to treat, or as-treated.<br>• If they had access to post trial treatment or potential confounding variables, please describe whether they included them in any time-varying analyses.<br>o Did the authors adjust for any covariates (time-varying or other)?<br>• Did the authors comment on any challenges they experienced with completing the follow-up study? (e.g. time or cost requirements). If so, please record with page number.<br>• Funders of follow-up study (if not stated, specify such).<br>o Did the follow-up study have industry sponsorship?<br>• Did the authors provide an estimate of the cost of the extension study? If so, record.

© 2018 Fitzpatrick T et al. *JAMA Network Open.*

eTable 3. Complete list of references for the 113 included extension studies and their corresponding original trial reports, according to outcome categorization†

Extension Study Reference	Original Trial Reference
**Statistically significant superiority of one treatment observed in trial extension phase**	
Aasa M, Dellborg M, Herlitz J, Svensson L, Grip L. Risk reduction for cardiac events after primary coronary intervention compared with thrombolysis for acute ST-elevation myocardial infarction (five-year results of the Swedish early decision reperfusion strategy [SWEDES] trial). Am J Cardiol 2010; 106(12): 1685-91.	Svensson L, Aasa M, Dellborg M, Gibson CM, Kirtane A, Herlitz J, et al. Comparison of very early treatment with either fibrinolysis or percutaneous coronary intervention facilitated with abciximab with respect to ST recovery and infarct-related artery epicardial flow in patients with acute ST-segment elevation myocardial infarction: The Swedish Early Decision (SWEDES) reperfusion trial. Am Heart J 2006; 151(4): 798.e1-7.
Alehagen U, Aaseth J, Johansson P. Reduced Cardiovascular Mortality 10 Years after Supplementation with Selenium and Coenzyme Q10 for Four Years: Follow-Up Results of a Prospective Randomized Double-Blind Placebo-Controlled Trial in Elderly Citizens, PLoS One 2015; 10(12): e0141641.	Alehagen U, Johansson P, Björnstedt M, Rosén A, Dahlström U. Cardiovascular mortality and N-terminal-proBNP reduced after combined selenium and coenzyme Q10 supplementation: A 5-year prospective randomized double-blind placebo-controlled trial among elderly Swedish citizens. Int J Cardiol 2013; 167: 1860–6.
Arbel Y, Klempfner R, Erez A, Goldenberg I, Benzekry S, Shlomo N, et al. Bezafibrate for the treatment of dyslipidemia in patients with coronary artery disease: 20-year mortality follow-up of the BIP randomized control trial. Cardiovasc Diabetol 2016; 15:11.	The Bezafibrate Infarction Prevention (BIP) Study. Secondary Prevention by Raising HDL Cholesterol and Reducing Triglycerides in Patients with Coronary Artery Disease the Bezafibrate Infarction Prevention (BIP) Study. Circulation 2000; 102: 21-7
Arnsrud Godtman R, Holmberg E, Lilja H, Stranne J, Hugosson J. Opportunistic Testing Versus Organized Prostate-specific Antigen Screening: Outcome After 18 Years in the Goteborg Randomized Population-based Prostate Cancer Screening Trial. Eur Urol 2015; 68(3): 354-60.	Hugosson J, Carlsson S, Aus G, Bergdahl S, Khatami A, Lodding P, et al. Mortality results from the Göteborg randomised population-based prostate-cancer screening trial. Lancet Oncol 2010; 11(8): 725–32
Aronson NE, Santosham M, Comstock GW, Howard RS, Moulton LH, Rhoades ER, Harrison LH. Long-term efficacy of BCG vaccine in American Indians and Alaska Natives: A 60-year follow-up study. JAMA 2004; 291(17): 2086-91.	Aronson JD and Palmer CE. Experience with BCG Vaccine in the Control of Tuberculosis among North American Indians. Public Health Rep 1946; 61(23): 802-20.
Arriagada R , Johansson H, Johansson U, Fornander T, Bergh J. Adjuvant radiotherapy in breast cancer: Results of the Stockholm randomised trial with 30-years of follow-up. European Society for Radiotherapy and Oncology 2010 Meeting Abstract.	Rutqvist LE, Cedermark B, Glas U, Johansson H, Rotstein S, Skoog L, et al. Radiotherapy, chemotherapy, and tamoxifen as adjuncts to surgery in early breast cancer: a summary of three randomized trials. Int J Radiat Oncol Biol Phys 1989; 16(3): 629-39.

© 2018 Fitzpatrick T et al. *JAMA Network Open.*

Extension Study Reference	Original Trial Reference
Austin J, Williams WR, Ross L, Hutchison S. Five-year follow-up findings from a randomized controlled trial of cardiac rehabilitation for heart failure. Eur J Cardiovasc Prev Rehabil 2008; 15(2): 162-7.	Austin J, Williams R, Ross L, Moseley L, Hutchison S. Randomised controlled trial of cardiac rehabilitation in elderly patients with heart failure. Eur J Heart Fail 2005; 7: 411-7
Bjurstam N, Björneld L, Warwick J, Sala E, Duffy SW, Nyström L, et al. The Gothenburg Breast Screening Trial: First Results on Mortality, Incidence, and Mode of Detection for Women Ages 39–49 Years at Randomization . Cancer 2003; 97(10): 2387-96.	Bjurstam N, Björneld L, Duffy SW, Smith TC, Cahlin E, Eriksson O, et al. The Gothenburg Breast Screening Trial First Results on Mortality, Incidence, and Mode of Detection for Women Ages 39–49 Years at Randomization. Cancer 1997; 80(11): 2091-9.
Bogner HR, Morales KH, Post EP, Bruce ML. Diabetes, depression, and death: a randomized controlled trial of a depression treatment program for older adults based in primary care (PROSPECT). Diabetes Care 2007; 30(12): 3005-10.	Bruce ML, Ten Have TR, Reynolds CF 3rd, Katz II, Schulberg HC, Mulsant BH, et al. Reducing Suicidal Ideation and Depressive Symptoms in Depressed Older Primary Care Patients. A Randomized Controlled Trial. JAMA. 2004; 291(9): 1081-91.
Buchwald H, Williams SE, Matts JP, Nguyen PA, Boen JR. Overall mortality in the program on the surgical control of the hyperlipidemias. J Am Coll Surg 2002; 195(3): 327-31.	Buchwald H, Matts JP, Fitch LL, Campos CT, Sanmarco ME, Amplatz K, et al Changes in sequential coronary arteriograms and subsequent coronary events. Surgical Control of the Hyperlipidemias (POSCH) Group. JAMA 1992; 268(11):1429-33.
Danish Breast Cancer Cooperative Group, Nielsen HM, Overgaard M, Grau C, Jensen AR, Overgaard J. Study of failure pattern among high-risk breast cancer patients with or without postmastectomy radiotherapy in addition to adjuvant systemic therapy: long-term results from the Danish Breast Cancer Cooperative Group DBCG 82 b and c randomized studies. J Clin Oncol 2006; 24(15): 2268-75.	Overgaard M, Hansen PS, Overgaard J, Rose C, Andersson M, Bach F, et al. Postoperative radiotherapy in high-risk premenopausal women with breast cancer who receive adjuvant chemotherapy. N Engl J Med 1997; 337(14): 949-55.
Davies WR, Brown AJ, Watson W, McCormick LM, West NE, Dutka DP, Hoole SP. Remote ischemic preconditioning improves outcome at 6 years after elective percutaneous coronary intervention: the CRISP stent trial long-term follow-up. Circ Cardiovasc Interv 2013; 6(3):246-51.	Hoole SP, Heck PM, Sharples L, Khan SN, Duehmke R, Densem CG, et al. Cardiac Remote Ischemic Preconditioning in Coronary Stenting (CRISP Stent) Study. A Prospective, Randomized Control Trial. Circulation 2009; 119 (6): 820-7.
Dolan KA, Shearer J, White B, Zhou J, Kaldor J, Wodak AD. Four-year follow-up of imprisoned male heroin users and methadone treatment: mortality, re-incarceration and hepatitis C infection. Addiction 2005; 100(6): 820-8.	Dolan KA, Shearer J, MacDonald M, Mattick RP, Hall W, Wodak AD. A randomised controlled trial of methadone maintenance treatment versus wait list control in an Australian prison system. Drug Alcohol Depend 2003; 72: 59-65.
Ekholm M, Bendahl PO, Fernö M, Nordenskjöld B, Stål O, Rydén L. Two years of adjuvant tamoxifen provides a survival benefit compared with no systemic treatment in premenopausal patients with primary breast cancer: Long-Term follow-up (> 25 years) of the phase III SBII:2pre trial. J Clin Oncol 2016; 34(19): 2232-8.	Rydén L, Jönsson PE, Chebil G, Dufmats M, Fernö M, Jirström K, et al. Two years of adjuvant tamoxifen in premenopausal patients with breast cancer: a randomised, controlled trial with long-term follow-up. Eur J Cancer 2005; 41(2): 256–64.

© 2018 Fitzpatrick T et al. *JAMA Network Open.*

Extension Study Reference	Original Trial Reference
Flossmann E, Rothwell PM; British Doctors Aspirin Trial and the UK-TIA Aspirin Trial. Effect of aspirin on long-term risk of colorectal cancer: consistent evidence from randomised and observational studies. Lancet 2007; 369: 1603-13.	**UK TIA Study:** Farrell B, Godwin J, Richards S, Warlow C. The United Kingdom transient ischaemic attack (UK-TIA) aspirin trial: final results. J Neurol Neurosurg Psychiatry 1991; 54(12): 1044-54. **British Doctors Aspirin Study:** Peto R, Gray R, Collins R, Wheatley K, Hennekens C, Jamrozik K, et al. Randomised trial of prophylactic daily aspirin in British male doctors. BMJ 1988; 296 (6618): 313-6.
Folkesson J, Birgisson H, Pahlman L, Cedermark B, Glimelius B, Gunnarsson U. Swedish Rectal Cancer Trial: long lasting benefits from radiotherapy on survival and local recurrence rate. J Clin Oncol 2005; 23(24): 5644-50.	Pahlman L. Initial report from a Swedish multicentre study examining the role of preoperative irradiation in the treatment of patients with resectable rectal carcinoma. Br J Surg 1993; 80(10);1333-6.
Ford I, Murray H, McCowan C, Packard CJ. Long-term safety and efficacy of lowering low-density lipoprotein cholesterol with statin therapy 20-year follow-up of west of Scotland coronary prevention study. Circulation 2016; 133(11): 1073-80.	Shepherd J, Cobbe SM, Ford I, Isles CG, Lorimer AR, MacFarlane PW, et al. Prevention of coronary heart disease with pravastatin in men with hypercholesterolemia. N Engl J Med 1995; 333(20):1301-7.
Frasure-Smith N, Prince R. Long-term follow-up of the Ischemic Heart Disease Life Stress Monitoring Program. Psychosom Med 1989; 51(5): 485-513.	Frasure-Smith N and Prince R. The Ischemic Heart Disease Life Stress Monitoring Program: Impact on Mortality. Psychosom Med 1985; 47(5):431-45.
Fritz D, Brouwer MC, van de Beek D. Dexamethasone and long-term survival in bacterial meningitis. Neurology 2012; 79(22): 2177-9.	de Gans J, van de Beek D; European Dexamethasone in Adulthood Bacterial Meningitis Study Investigators. Dexamethasone in adults with bacterial meningitis. N Engl J Med 2002; 347(20): 1549-56.
Gitlin LN, Hauck WW, Dennis MP, Winter L, Hodgson N, Schinfeld S. Long-term effect on mortality of a home intervention that reduces functional difficulties in older adults: results from a randomized trial. J Am Geriatr Soc 2009; 57(3): 476-81.	Gitlin LN, Winter L, Dennis MP, Corcoran M, Schinfeld S, Hauck WW. A Randomized Trial of a Multicomponent Home Intervention to Reduce Functional Difficulties in Older Adults. J Am Geriatr Soc 2006; 54 (5): 809–16.
Gjestad R, Franck J, Lindberg S, Haver B. Early treatment for Women with Alcohol Addiction (EWA) reduces mortality: A randomized controlled trial with long-term register follow-up. Alcohol Alcohol 2011; 46(2): 170-6.	Dahlgren L and Willander A. Are Special Treatment Facilities for Female Alcoholics Needed? A Controlled 2-Year Follow-up Study from a Specialized Female Unit (EWA) Versus a Mixed Male/Fernale Treatment Facility. Alcohol Clin Exp Res 1989; 13(4): 499-504.
Goodin DS, Reder AT, Ebers GC, Cutter G, Kremenchutzky M, Oger J, et al. Survival in MS: A randomized cohort study 21 years after the start of the pivotal IFN -1b trial. Neurology 2012; 78: 1315-22.	Duquette P, Despault L, Knobler L, Lublin FD, Kelley L, Francis GS, et al. Interferon beta-lb in the treatment of multiple sclerosis: Final outcome of the randomized controlled trial. Neurology 1995; 45(7): 1277-85.
Hall AS, Murray GD, Ball SG. Follow-up study of patients randomly allocated ramipril or placebo for heart failure after acute myocardial infarction: AIRE Extension (AIREX) Study. Lancet 1997; 349: 1493-7.	The Acute Infarction Ramipril Efficacy (AIRE) Study Investigators. Effect of ramipril on mortality and morbidity of survivors of acute myocardial infarction with clinical evidence of heart failure. Lancet 1993; 342: 821-28.

© 2018 Fitzpatrick T et al. *JAMA Network Open.*

Extension Study Reference	Original Trial Reference
Hansen JM, Wildner-Christensen M, Hallas J, Schaffalitzky de Muckadell OB. Effect of a Community Screening for Helicobacter pylori: A 5-Year Follow-Up Study. Am J Gastroenterol 2008; 103(5): 1106-13.	Wildner-Christensen M, Møller Hansen J, Schaffalitzky De Muckadell OB. Rates of Dyspepsia One Year After Helicobacter pylori Screening and Eradication in a Danish Population. Gastroenterology 2003; 125(2): 372–9.
Heart Protection Study Collaborative Group. Effects on 11-year mortality and morbidity of lowering LDL cholesterol with simvastatin for about 5 years in 20 536 high-risk individuals: a randomised controlled trial. Lancet 2011; 378: 2013-20.	Collins R, Armitage J, Parish S, Sleigh P, Peto R; Heart Protection Study Collaborative Group. MRC/BHF Heart Protection Study of cholesterol-lowering with simvastatin in 5963 people with diabetes: a randomised placebo-controlled trial. Lancet 2003; 361: 2005–16.
Holm T, Singnomklao T, Rutqvist LE, Cedermark B. Adjuvant preoperative radiotherapy in patients with rectal carcinoma: Adverse effects during long term follow-up of two randomized trials. Cancer 1996; 78(5): 968-76.	Stockholm Rectal Cancer Study Group. Preoperative Short-term Radiation Therapy in Operable Rectal Carcinoma. A Prospective Randomized Trial. Cancer 1990; 66: 49-56.
Holme Ø, Løberg M, Kalager M, Bretthauer M, Hernán MA, Aas E, et al. Effect of flexible sigmoidoscopy screening on colorectal cancer incidence and mortality: a randomized clinical trial. JAMA 2014; 312(6): 606-15.	Hoff G, Grotmol T, Skovlund E, Bretthauer M; the Norwegian Colorectal Cancer Pevention Study Group. Risk of colorectal cancer seven years after flexible sigmoidoscopy screening: randomised controlled trial. BMJ 2009;338: b1846
Hulse GK, Tait RJ. Five-year outcomes of a brief alcohol intervention for adult in-patients with psychiatric disorders. Addiction 2003; 98(8): 1061-8.	Hulse GK and Tait RJ. Six-month outcomes associated with a brief alcohol intervention for adult in-patients with psychiatric disorders. Drug Alcohol Rev 2002; 21: 105-12.
Jansen AF, Rijneveld WJ, Remeijer L, Völker-Dieben HJ, Eggink CA, Geerards AJ, et al. Five-Year Follow-Up on the Effect of Oral Acyclovir After Penetrating Keratoplasty for Herpetic Keratitis. Cornea 2009; 28(8): 843-5.	van Rooij J, Rijneveld WJ, Remeijer L, Völker-Dieben HJ, Eggink CA, Geerards AJ, et al. Effect of Oral Acyclovir after Penetrating Keratoplasty for Herpetic Keratitis. A Placebo-Controlled Multicenter Trial. Ophthalmology 2003; 110(10): 1916–9.
Kim LG, P Scott RA, Ashton HA, Thompson SG; Multicentre Aneurysm Screening Study Group. A sustained mortality benefit from screening for abdominal aortic aneurysm. Ann Intern Med 2007; 146(10): 699-706.	Ashton HA, Buxton MJ, Day NE, Kim LG, Marteau TM, Scott RA, et al. The Multicentre Aneurysm Screening Study (MASS) into the effect of abdominal aortic aneurysm screening on mortality in men: a randomised controlled trial. Lancet 2002; 360: 1531–39
Knatterud GL, Rosenberg Y, Campeau L, Geller NL, Hunninghake DB, Forman SA, et al. Long-term effects on clinical outcomes of aggressive lowering of low- density lipoprotein cholesterol levels and low-dose anticoagulation in the post coronary artery bypass graft trial. Circulation 2000; 102(2): 157-65.	The Post Coronary Artery Bypass Graft Trial Investigators. The effect of aggressive lowering of low-density lipoprotein cholesterol levels and low-dose anticoagulation on obstructive changes in saphenous-vein coronary-artery bypass grafts. N Engl J Med 1997; 336(3):153-62.
Koh KK. Long-term effectiveness and safety of pravastatin in patients with coronary heart disease : 16 Years of Follow-Up of the LIPID Study. Circulation 2016; 134(13): e294-5.	The Long-Term Intervention with Pravastatin in Ischaemic Disease (LIPID) Study Group. Prevention of cardiovascular events and death with pravastatin in patients with coronary heart disease and a broad range of initial cholesterol levels. N Engl J Med 1998; 339(19): 1349-57.

© 2018 Fitzpatrick T et al. *JAMA Network Open.*

Extension Study Reference	Original Trial Reference
Kostis JB, Cabrera J, Cheng JQ, Cosgrove NM, Deng Y, Pressel SL, Davis BR. Association Between Chlorthalidone Treatment of Systolic Hypertension and Long-term Survival. JAMA 2011; 306(23): 2588-93.	SHEP Cooperative research group. Prevention of Stroke by Antihypertensive Drug Treatment in Older Persons with Isolated Systolic Hypertension Final Results of the Systolic Hypertension in the Elderly Program (SHEP). JAMA 1991; 265(24): 3255-65.
Küchler T, Bestmann B, Rappat S, Henne-Bruns D, Wood-Dauphinee S. Impact of psychotherapeutic support for patients with gastrointestinal cancer undergoing surgery: 10-Year survival results of a randomized trial. J Clin Oncol 2007; 25(19): 2702-8.	Kuchler T, Henne-Bruns D, Rappat S, Graul J, Holst K, Williams JI, Wood-Dauphinee S. Impact of psychotherapeutic support on gastrointestinal cancer patients undergoing surgery: survival results of a trial. Hepatogastroenterology 1999; 46(25): 322-35.
Labrie F, Candas B, Cusan L, Gomez JL, Bélanger A, Brousseau G, Chevrette E, Lévesque J. Screening decreases prostate cancer mortality: 11-year follow-up of the 1988 Quebec prospective randomized controlled trial. Prostate 2004; 59(3): 311-8.	Labrie F, Candas B, Dupont A, Cusan L, Gomez JL, Suburu RE, et al. Screening Decreases Prostate Cancer Death: First Analysis of the 1988 Quebec Prospective Randomized Controlled Trial. Prostate 1999; 38(2): 83–91.
Lim WH, Russ GR, Wong G, Pilmore H, Kanellis J, Chadban SJ. The risk of cancer in kidney transplant recipients may be reduced in those maintained on everolimus and reduced cyclosporine. Kidney Int 2017; 91(4): 954-63.	Tedesco Silva H, Cibrik D, Johnston T, Lackova E, Mange K, Panis C, et al. Everolimus Plus Reduced-Exposure CsA versus Mycophenolic Acid Plus Standard-Exposure CsA in Renal-Transplant Recipients. Am J Transplant 2010; 10: 1401-13.
Lloyd SM, Stott DJ, de Craen AJ, Kearney PM, Sattar N, Perry I, et al. Long-term effects of statin treatment in elderly people: extended follow-up of the PROspective Study of Pravastatin in the Elderly at Risk (PROSPER). PLoS One 2013; 8(9): e72642.	Shepherd J, Blauw GJ, Murphy MB, Bollen EL, Buckley BM, Cobbe SM, et al. Pravastatin in elderly individuals at risk of vascular disease (PROSPER): a randomised controlled trial. Lancet 2002; 360: 1623–30.
Lundström H, Siersma V, Nielsen AB, Brodersen J, Reventlow S, Andersen PK, de Fine Olivarius N. Structured personal care of type 2 diabetes: a 19 year follow-up of the study Diabetes Care in General Practice (DCGP). Diabetologia 2014; 57(6): 1119-23.	de Fine Olivarius N, Beck-Nielsen H, Andreasen AH, Hørder M, Pedersen PA. Randomised controlled trial of structured personal care of type 2 diabetes mellitus. BMJ 2001; 323: 1-9.
Maas AC, van Domburg RT, Deckers JW, Vermeer F, Remme WJ, Kamp O. Sustained benefit at 10-14 years follow-up after thrombolytic therapy in myocardial infarction. Eur Heart J 1999; 20(11): 819-26.	Simoons ML, Serruys PW, vd Brand M, Bär F, de Zwaan C, Res J, et al. Improved survival after early thrombolysis in acute myocardial infarction. A Randomised Trial by the Interuniversity Cardiology Institute in The Netherlands. Lancet 1985; 326(8455): 578-81.
Menon V, Li L, Wang X, Greene T, Balakrishnan V, Madero M, et al. Adiponectin and mortality in patients with chronic kidney disease. J Am Soc Nephrol 2006; 17(9): 2599-606.	Peterson JC, Adler S, Burkart JM, Greene T, Hebert LA, Hunsicker LG, et al. Blood pressure control, proteinuria, and the progression of renal disease. The Modification of Diet in Renal Disease Study. Ann Intern Med 1995; 123: 754-62.
Molyneux AJ, Birks J, Clarke A, Sneade M, Kerr RS. The durability of endovascular coiling versus neurosurgical clipping of ruptured cerebral aneurysms: 18 year follow-up of the UK cohort of the International Subarachnoid Aneurysm Trial (ISAT). Lancet 2015; 385: 691-7.	Molyneux AJ, Kerr RS, Yu LM, Clarke M, Sneade M, Yarnold JA, et al. International Subarachnoid Aneurysm Trial (ISAT) of neurosurgical clipping versus endovascular coiling in 2143 patients with ruptured intracranial aneurysms: a randomised trial. Lancet 2002; 360: 1267–74.

© 2018 Fitzpatrick T et al. *JAMA Network Open.*

Extension Study Reference	Original Trial Reference
Nielsen PH, Terkelsen CJ, Nielsen TT, Thuesen L, Krusell LR, Thayssen P, et al. System Delay and Timing of Intervention in Acute Myocardia Infarction (from the Danish Acute Myocardial Infarction-2 [DANAMI-2] Trial). Am J Cardiol 2011; 108(6): 776-81.	Andersen HR, Nielsen TT, Rasmussen K, Thuesen L, Kelbaek H, Thayssen P, et al. Comparison of Coronary Angioplasty with Fibrinolytic Therapy in Acute Myocardial Infarction. N Engl J Med 2003; 349(8): 733-44.
Nyström L, Andersson I, Bjurstam N, Frisell J, Nordenskjöld B, Rutqvist LE. Long-term effects of mammography screening: updated overview of the Swedish randomised trials. Lancet 2002; 359: 909-19.	**Malmo:** Andersson I, Aspegren K, Janzon L, Landberg T, Lindholm K, Linell F, et al. Mammographic screening and mortality from breast cancer: the Malmo mammographic screening trial. BMJ 1988;297: 943-8. **Two-Country:** Tabár L, Fagerberg CJ, Gad A, Baldetorp L, Holmberg LH, Gröntoft O, et al. Reduction in mortality from breast cancer after mass screening with mammography. Randomised Trial from the Breast Cancer Screening Working Group of the Swedish National Board of Health and Welfare. Lancet 1985; 8433: 829-33. **Stockholm Trial:** Frisell J, Glas U, Hellstrom L, Somell A. Randomized mammographic screening for breast cancer in Stockholm Design, first round results and comparisons. Breast Cancer Res Treat 1986; 8 (1): 45-54. **Goteborg:** Bjurstam N, Björneld L, Duffy SW, Smith TC, Cahlin E, Eriksson O, et al. The Gothenburg Breast Screening Trial First Results on Mortality, Incidence, and Mode of Detection for Women Ages 39–49 Years at Randomization. Cancer 1997; 80(11): 2091–9.
Olds DL, Kitzman H, Knudtson MD, Anson E, Smith JA, Cole R. Effect of home visiting by nurses on maternal and child mortality: Results of a 2-Decade follow-up of a randomized clinical trial. JAMA Pediatr 2014; 168(9): 800-6.	Kitzman H, Olds DL, Henderson CR Jr, Hanks C, Cole R, Tatelbaum R, et al. Effect of Prenatal and Infancy Home Visitation by Nurses on Pregnancy Outcomes, Childhood Injuries, and Repeated Childbearing. A Randomized Controlled Trial. JAMA. 1997;278(8): 644-52.
Plüss CE, Billing E, Held C, Henriksson P, Kiessling A, Karlsson MR, Wallen HN. Long-term effects of an expanded cardiac rehabilitation programme after myocardial infarction or coronary artery bypass surgery: a five-year follow-up of a randomized controlled study. Clin Rehabil 2011; 25(1): 79-87.	Plüss CE, Karlsson MR, Wallen NH, Billing E, Held C. Effects of an expanded cardiac rehabilitation programme in patients treated for an acute myocardial infarction or a coronary artery by-pass graft operation. Clinical Rehabil 2008;22(4):306-18.
Puolakka K, Kautiainen H, Möttönen T, Hannonen P, Korpela M, Julkunen H, et al. Impact of Initial Aggressive Drug Treatment with a Combination of Disease-Modifying Antirheumatic Drugs on the Development of Work Disability in Early Rheumatoid Arthritis: A Five-Year Randomized Followup Trial. Arthritis Rheum 2004;50(1):55-62.	Möttönen T, Hannonen P, Leirisalo-Repo M, Nissilä M, Kautiainen H, Korpela M, et al. Comparison of combination therapy with single-drug therapy in early rheumatoid arthritis: a randomised trial. FIN-RACo trial group. Lancet 1999; 353: 1568 – 73.
Qu C, Chen T, Fan C, Zhan Q, Wang Y, Lu J, et al. Efficacy of Neonatal HBV Vaccination on Liver Cancer and Other Liver	Sun Z, Zhu Y, Stjernsward J, Hilleman M, Collins R, Zhen Y, et al. Design and compliance of HBV vaccination trial on newborns to prevent hepatocellular

© 2018 Fitzpatrick T et al. *JAMA Network Open.*

Extension Study Reference	Original Trial Reference
Diseases over 30-Year Follow-up of the Qidong Hepatitis B Intervention Study: A Cluster Randomized Controlled Trial. PLoS Med 2014; 11(12): e1001774.	carcinoma and 5-year results of its pilot study. Cancer Detect Prev. 1991; 15(4): 313-8.
Rana MM, Huhtala H, Apter D, Eriksson T, Luostarinen T, Natunen K, et al. Understanding long-term protection of human papillomavirus vaccination against cervical carcinoma: Cancer registry-based follow-up. Int J Cancer 2013; 132(12): 2833-8.	The FUTURE II Study Group. Quadrivalent Vaccine against Human Papillomavirus to Prevent High-Grade Cervical Lesions. N Engl J Med 2007; 356(19): 1915-27.
Rathsman B, Donner M, Ursing C, Nyström T. Long-term effects of intensive treatment in type 1 diabetes on all-cause mortality, cardiovascular mortality, and morbidity in cardiovascular disease: A long-term follow-up study. Pediatr Diabetes 2013; 14: 24.	Reichard P, Britz A, Cars I, Nilsson BY, Sobocinsky-Olsson B, Rosenqvist U. The Stockholm Diabetes Intervention Study (SDIS): 18 Months' Results. Acta Med Scand 1988; 224 (2): 115-22.
Ronco G, Dillner J, Elfström KM, Tunesi S, Snijders PJ, Arbyn M, et al. Efficacy of HPV-based screening for prevention of invasive cervical cancer: Follow-up of four European randomised controlled trials. Lancet 2014; 383: 524-32.	**Swedescreen:** Forslund O, Antonsson A, Edlund K, van den Brule AJ, Hansson BG, Meijer CJ, et al. Population-based type-specific prevalence of high-risk human papillomavirus infection in middle-aged Swedish Women. J Med Virology 2002; 66: 535-41.<br>**POBASCAM**: Bulkmans NW, Rozendaal L, Snijders PJ, Voorhorst FJ, Boeke AJ, Zandwijken GR, et al. Pobascam, a population-based randomized controlled trial for implementation of high-risk HPV testing in cervical screening: design, methods and baseline data of 44,102 women. Int. J. Cancer 2004; 110: 94–101.<br>**ARTISTIC**: Kitchener HC, Almonte M, Wheeler P, Desai M, Gilham C, Bailey A, et al. HPV testing in routine cervical screening: cross sectional data from the ARTISTIC trial. Br J Cancer 2006; 95: 56 – 61.<br>**NTCC**: Ronco G, Giorgi-Rossi P, Carozzi F, Confortini M, Dalla Palma P, Del Mistro A, et al. Efficacy of human papillomavirus testing for the detection of invasive cervical cancers and cervical intraepithelial neoplasia: a randomised controlled trial. Lancet Oncol 2010; 11: 249–57.
Rosell J, Nordenskjöld B, Bengtsson NO, Fornander T, Hatschek T, Lindman H, et al. Effects of adjuvant tamoxifen therapy on cardiac disease: results from a randomized trial with long-term follow-up. Breast Cancer Res Treat 2013; 138(2): 467-73.	Nordenskjöld B, Rosell J, Rutqvist LE, Malmström PO, Bergh J, Bengtsson NO, et al. Coronary Heart Disease Mortality After 5 Years of Adjuvant Tamoxifen Therapy: Results from a Randomized Trial. J Natl Cancer Inst 2005; 97(21):1609 – 10.
Rousseau MF, Jong P, Ahn SA, Pouleur H. Long-term effect of early enalapril therapy on causes of death in patients with left ventricular dysfunction: A 27-year follow-up of SOLVD in Belgium. J Am Coll Cardiol 2016; 67(13): 1346.	SOLVD Investigators, Yusuf S, Pitt B, Davis CE, Hood WB, Cohn JN. Effect of Enalapril on survival in patients with reduced left ventricular ejection fractions and congestive heart failure. N Engl J Med 1991; 325(5): 293 - 303.

© 2018 Fitzpatrick T et al. *JAMA Network Open.*

Extension Study Reference	Original Trial Reference
Scholefield JH, Moss SM, Mangham CM, Whynes DK, Hardcastle JD. Nottingham trial of faecal occult blood testing for colorectal cancer: A 20-year follow-up. Gut 2012; 61(7): 1036-40.	Hardcastle JD, Chamberlain JO, Robinson MH, Moss SM, Amar SS, Balfour TW, et al. Randomised controlled trial of faecal-occult-blood screening for colorectal cancer. Lancet 1996; 348: 1472–7.
Schröder FH, Hugosson J, Roobol MJ, Tammela TL, Zappa M, Nelen V, et al. Screening and prostate cancer mortality: Results of the European Randomised Study of Screening for Prostate Cancer (ERSPC) at 13 years of follow-up. Lancet 2014; 384: 2027-35.	Schröder FH, Hugosson J, Roobol MJ, Tammela TL, Ciatto S, Nelen V, et al. Screening and Prostate-Cancer Mortality in a Randomized European Study. N Engl J Med 2009; 360: 1320-8.
Secher RG, Hjorthøj CR, Austin SF, Thorup A, Jeppesen P, Mors O, Nordentoft M. Ten-year follow-up of the OPUS specialized early intervention trial for patients with a first episode of psychosis. Schizophr Bull 2015; 41(3): 617-26.	Petersen L1, Jeppesen P, Thorup A, Abel MB, Øhlenschlaeger J, Christensen TØ, et al. A randomised multicentre trial of integrated versus standard treatment for patients with a first episode of psychotic illness. BMJ 2005; 331: 602.
Sever PS, Chang CL, Gupta AK, Whitehouse A, Poulter NR; ASCOT Investigators. The Anglo-Scandinavian Cardiac Outcomes Trial: 11-year mortality follow-up of the lipid-lowering arm in the U.K. Eur Heart J 2011; 32(10): 2525-32.	Sever PS, Poulter NR, Dahlöf B, Wedel H; Anglo-Scandinavian Cardiac Outcomes Trial Investigators. Different Time Course for Prevention of Coronary and Stroke Events by Atorvastatin in the Anglo-Scandinavian Cardiac Outcomes Trial–Lipid-lowering Arm (ASCOT-LLA). J Cardiol 2005; 96(S): 39F-44F.
Stamler J, Neaton JD, Cohen JD, Cutler J, Eberly L, Grandits G, et al. Multiple Risk Factor Intervention Trial Revisited: A New Perspective Based on Nonfatal and fatal Composite Endpoints, Coronary and Cardiovascular, During the Trial. J Am Heart Assoc 2012; 1(5): e003640.	Stamler J, Caggiula A, Grandits GA, Kjelsberg M, Cutler JA. Relationship to Blood Pressure of Combinations of Dietary Macronutrients: Findings of the Multiple Risk Factor Intervention Trial (MRFIT). Circulation 1996; 94(10): 2417-23.
Stewart JC, Perkins AJ, Callahan CM. Effect of collaborative care for depression on risk of cardiovascular events: data from the IMPACT randomized controlled trial. Psychosom Med 2014; 76(1): 29-37.	Unützer J, Katon W, Callahan CM, Williams JW Jr, Hunkeler E, Harpole L, et al. Collaborative care management of late-life depression in the primary care setting. A randomized controlled trial. JAMA 2002; 288(22): 2836-45.
Stewart S, Carrington MJ, Horowitz JD, Marwick TH, Newton PJ, Davidson PM, et al. Prolonged impact of home versus clinic-based management of chronic heart failure: Extended follow-up of a pragmatic, multicentre randomized trial cohort. Int J Cardiol 2014; 174(3): 600-10.	Stewart S, Carrington MJ, Marwick TH, Davidson PM, Macdonald P, Horowitz JD, et al. Impact of Home Versus Clinic-Based Management of Chronic Heart Failure The WHICH? (Which Heart Failure Intervention Is Most Cost-Effective & Consumer Friendly in Reducing Hospital Care) Multicenter, Randomized Trial. J Am Coll Cardiol 2012; 60 (14): 1239–48.
Strandberg TE, Pyörälä K, Cook TJ, Wilhelmsen L, Faergeman O, Thorgeirsson G, et al. Mortality and incidence of cancer during 10-year follow-up of the Scandinavian Simvastatin Survival Study (4S). Lancet 2004; 364: 771-7.	Scandinavian Simvastatin Survival Study (4S) Group. Randomised trial of cholesterol lowering in 4444 patients with coronary heart disease: the Scandinavian Simvastatin Survival Study (4S). Lancet 1994; 344: 1383-9.
Wallentin L, Lindhagen L, Ärnström E, Husted S, Janzon M, Johnsen SP, et al. Early invasive versus non-invasive treatment in	FRISC II Investigators. Invasive compared with non-invasive treatment in unstable coronary-artery disease: FRISC II prospective randomised multicentre

© 2018 Fitzpatrick T et al. *JAMA Network Open.*

Extension Study Reference	Original Trial Reference
patients with non-ST-elevation acute coronary syndrome (FRISC-II): 15 year follow-up of a prospective, randomised, multicentre study. Lancet 2016; 388: 1903-11.	study. FRagmin and Fast Revascularisation during InStability in Coronary artery disease Investigators. Lancet 1999; 354(9180): 708–15.
**Harms observed in trial extension phase**	
Ajeganova S, Svensson B, Hafström I; BARFOT Study Group. Low-dose prednisolone treatment of early rheumatoid arthritis and late cardiovascular outcome and survival: 10-year follow-up of a 2-year randomised trial. BMJ Open 2014; 4(4): e004259.	Svensson B1, Boonen A, Albertsson K, van der Heijde D, Keller C, Hafström I. Low -dose prednisolone in addition to the initial disease-modifying antirheumatic drug inpatients with early active rheumatoid arthritis reduces joint destruction and increases the remission rate: a two-year randomized trial. Arthritis Rheum 2005; 52(11):3360–70.
Heliövaara-Peippo S, Halmesmäki K, Hurskainen R, Teperi J, Grenman S, Kivelä A, et al. The effect of hysterectomy or levonorgestrel-releasing intrauterine system on lower urinary tract symptoms: a 10-year follow-up study of a randomised trial. BJOG 2010; 117(5): 602-9.	Hurskainen R, Teperi J, Rissanen P, Aalto AM, Grenman S, Kivelä A, et al. Quality of life and cost-effectiveness of levonorgestrel-releasing intrauterine system versus hysterectomy for treatment of menorrhagia: a randomised trial. Lancet 2001; 357: 273–7.
Hemminki E, Meriläinen J. Long-term follow-up of mothers and their infants in a randomized trial on iron prophylaxis during pregnancy. Am J Obstet Gynecol 1995; 173(1): 205-9.	Hemminki E and Rimpelä U. A randomized comparison of routine versus selective iron supplementation during pregnancy. J Am Coll Nutr 1991; 10(1): 3-10.
Jones JM, Ribeiro GG. Mortality Patterns over 34 years of Breast Cancer Patients in a Clinical Trial of Post-operative Radiotherapy. Clin Radiol 1989; 40(2): 204-8.	Cuzick J, Stewart H, Peto R, Baum M, Fisher B, Host H, et al. Overview of randomized trials of postoperative adjuvant radiotherapy in breast cancer. Cancer Treat Rep 1987;71(1):15-29.
Killander F, Anderson H, Kjellén E, Malmström P. Increased cardio and cerebrovascular mortality in breast cancer patients treated with postmastectomy radiotherapy - 25 year follow-up of a randomised trial from the South Sweden Breast Cancer Group. Eur J Cancer 2014; 50(13): 2201-10.	Ryden S, Ferno M, Moller T, Aspegren K, Bergljung L, Killander D, Landberg T. Long-Term Effects of Adjuvant Tamoxifen and/or Radiotherapy: The South Sweden Breast Cancer Trial. Acta Oncologica 1992; 31(2): 271-4.
Mortensen FV, Jensen LS, Sørensen HT, Pedersen L. Cause-specific mortality associated with leukoreduced, buffy coat-depleted, or no blood transfusion after elective surgery for colorectal cancer: a posttrial 15-year follow-up study. Transfusion 2011; 51(2): 259-63.	Jensen LS, Kissmeyer-Nielsen P, Wolff B, Qvist N. Randomised comparison of leucocyte-depleted versus buffy-coat-poor blood transfusion and complications after colorectal surgery. Lancet 1996; 348: 841–5.
Onsrud M, Lindemann K, Kristensen G, Trope CG. Survival after postoperative radiotherapy for early stage endometrial carcinoma: The Oslo study revisited after up to 43 years of follow-up. Int J Gynaecol Obstet 2012; 119: S444-5.	Aalders J, Abeler V, Kolstad P, Onsrud M. Postoperative external irradiation and prognostic parameters in stage I endometrial carcinoma: clinical and histopathologic study of 540 patients. Obstet Gynecol 1980; 56(4): 419-27.

© 2018 Fitzpatrick T et al. *JAMA Network Open.*

Extension Study Reference	Original Trial Reference
Perez MV, Wang PJ, Larson JC, Virnig BA, Cochrane B, Curb JD, et al. Effects of postmenopausal hormone therapy on incident atrial fibrillation: The women's health initiative randomized controlled trials. Circ Arrhythm Electrophysiol 2012; 5(6): 1108-16.	Rossouw JE, Anderson GL, Prentice RL, LaCroix AZ, Kooperberg C, Stefanick ML, et al. Risk and benefits of estrogen plus progestin in healthy postmenopausal women: principal results from the Women's Health Initiative Randomized Controlled Trial. JAMA 2002; 288(3): 321-34.
Persson PE, Nilsson OS, Berggren AM. Do non-steroidal anti-inflammatory drugs cause endoprosthetic loosening? A 10-year follow-up of a randomized trial on ibuprofen for prevention of heterotopic ossification after hip arthroplasty. Acta Orthop 2005; 76(6): 735-40.	Persson PE, Sodemann B, Nilsson OS. Preventive effects of ibuprofen on periarticular heterotopic ossification after total hip arthroplasty. A randomized double-blind prospective study of treatment time. Acta Orthop Scand 1998; 69 (2): 111-5
Radford L, Bolland MJ, Mason B, Horne AM, Grey A, Gamble GD, Reid IR. Adverse cardiovascular effects of calcium supplements may not persist after discontinuation of supplements: 5-year follow up of the Auckland calcium study. Osteoporosis Int 2011; 22(1).	Reid IR, Mason B, Horne A, Ames R, Reid HE, Bava U, et al. Randomized Controlled Trial of Calcium in Healthy Older Women. Am J Med 2006; 119: 777-85.
Vessey MP, Fairweather DV, Norman-Smith B, Buckley J. A randomized double-blind controlled trial of the value of stilboestrol therapy in pregnancy: long-term follow-up of mothers and their offspring. Br J Obstet Gynaecol 1983; 90(11):1007-17.	Swyer GIM and Law RG. An evaluation of the prophylactic antenatal use of stilboestrol: Preliminary report. J Endocrinol 1954;10:6-7.
von Bormann B, Suksompong S, Schleinzer W. Blood transfusions and prognosis in colorectal cancer: long-term results of a randomized controlled trial. Ann Surg 2015; 261(5): e136.	Busch OR, Hop WC, Hoynck van Papendrecht MA, Marquet RL, Jeekel J. Blood transfusions and prognosis in colorectal cancer. N Engl J Med 1993; 328(19): 1372-6.
Winkel P, Hilden J, Hansen JF, Kastrup J, Kolmos HJ, Kjøller E, et al. Clarithromycin for stable coronary heart disease increases all-cause and cardiovascular mortality and cerebrovascular morbidity over 10 years in the CLARICOR randomised, blinded clinical trial. Int J Cardiol 2015; 182: 459-65.	Jespersen CM, Als-Nielsen B, Damgaard M, Fischer Hanser J, Hansen S, Help OH, et al. Randomised placebo controlled multicentre trial to assess short term clarithromycin for patients with stable coronary heart disease: CLARICOR trial. BMJ 2000; 332: 226.
**Null effects in original trial also seen in the trial extension phase**	
Appel JM, Zerahn B, Møller S, Christensen HM, Søgaard P, Ejlertsen B, et al. Long-term heart function after adjuvant epirubicin chemotherapy for breast cancer. Acta Oncol 2012; 51(8): 1054-61.	Ejlertsen B, Mouridsen HT, Jensen MB, Andersen J, Cold S, Edlund P, et al. Improved outcome from substituting methotrexate with epirubicin: Results from a randomised comparison of CMF versus CEF in patients with primary breast cancer. Eur J Cancer 2007; 43: 877-84.
Beebe GW, Simon AH, Vivona S. Long term mortality follow up of army recruits who received adjuvant influenza virus vaccine in 1951-1853. Am J Epidemiol 1972; 95(4): 337-46.	Beebe GW, Simon A, Vivona S. Follow-up study on army personnel who received adjuvant influenza virus vaccine 1951-1953. Am J Med Sci 1964; 247: 385-406.

© 2018 Fitzpatrick T et al. *JAMA Network Open.*

Extension Study Reference	Original Trial Reference
Brouwers FP, Asselbergs FW, Hillege HL, de Boer RA, Gansevoort RT, van Veldhuisen DJ, van Gilst WH. Long-term effects of fosinopril and pravastatin on cardiovascular events in subjects with microalbuminuria: Ten years of follow-up of Prevention of Renal and Vascular End-stage Disease Intervention Trial (PREVEND IT). Am Heart J 2011;161(6): 1171-8.	Asselbergs FW, Diercks GF, Hillege HL, van Boven AJ, Janssen WM, Voors AA, et al. Effects of Fosinopril and Pravastatin on Cardiovascular Events in Subjects with microalbuminuria. Circulation. 2004; 110(18): 2809-16.
Cherry N, McNamee R, Heagerty A, Kitchener H, Hannaford P. Long-term safety of unopposed estrogen used by women surviving myocardial infarction: 14-year follow-up of the ESPRIT randomised controlled trial. BJOG 2014; 121(6): 700-5.	Cherry N, Gilmour K, Hannaford P, Heagerty A, Khan MA, Kitchener H, et al. Oestrogen therapy for prevention of reinfarction in postmenopausal women: a randomised placebo controlled trial. Lancet 2002; 360: 2001–8.
Cook NR, Appel LJ, Whelton PK. Sodium Intake and All-Cause Mortality Over 20 Years in the Trials of Hypertension Prevention. J Am Coll Cardiol 2016; 68(15): 1609-17.	The Trials of Hypertension Prevention Collaborative Research Group. The Effects of Nonpharmacologic Interventions on Blood Pressure of Persons with High Normal Levels. Results of the Trials of Hypertension Prevention, Phase I. JAMA 1992;267: 1213-20.
Cushman WC, Davis BR, Pressel SL, Cutler JA, Einhorn PT, Ford CE, et al. Mortality and Morbidity During and After the antihypertensive and Lipid-Lowering Treatment to Prevent Heart Attack Trial. J Clin Hypertens (Greenwich) 2012; 14(1): 20-31.	ALLHAT Collaborative Research Group. Diuretic Versus Alpha-Blocker as First-Step Antihypertensive Therapy. Final Results from the Antihypertensive and Lipid-Lowering Treatment to Prevent Heart Attack Trial (ALLHAT). Hypertension 2003; 42(3):239-46.
Delaney EK, Murchie P, Lee AJ, Ritchie LD, Campbell NC. Secondary prevention clinics for coronary heart disease: a 10-year follow-up of a randomised controlled trial in primary care. Heart 2008; 94(11): 1419-23.	Campbell N, Ritchie L, Thain J, Deans H, Rawles J, Squair J. Secondary prevention in coronary heart disease: baseline survey of provision in general practice. BMJ 1998; 80(5): 447-52.
Gao YC, Chen J, Qin Q, Chen H, Wang W, Zhao J, et al. Long-term follow-up of a randomized trial of laparascopic cholecystectomy and laparascopic common bile duct exploration vs. ERCP with sphincterotomy and laparascopic cholecystectomy for common bile duct stone disease. Medicine 2017; 96(37): e7925.	Rogers SJ, Cello JP, Horn JK, Siperstein AE, Schecter WP, Campbell AR, et al. Prospective Randomized Trial of LC+ LCBDE vs ERCP/S+ LC for Common Bile Duct Stone Disease. Arch Surg. 2010;145(1):28-33.
Gårdmark T, Jahnson S, Wahlquist R, Wijkström H, Malmström PU. Analysis of progression and survival after 10 years of a randomized prospective study comparing mitomycin-C and bacillus Calmette-Guerin in patients with high-risk bladder cancer. BJU Int 2007; 99(4): 817-20.	Lundholm C, Norlén BJ, Ekman P, Jahnson S, Lagerkvist M, Lindeborg T, et al. A randomized prospective study comparing long-term intravesical instillations of mitomycin C and bacillus Calmette-Guerin in patients with superficial bladder carcinoma. J Urology 1996; 156: 372-6.
Gibson A, Degenhardt L, Mattick RP, Ali R, White J, O'Brien S. Exposure to opioid maintenance treatment reduces long-term mortality. Addiction 2008; 103(3): 462-8.	Mattick RP, Ali R, White JM, O'Brien S, Wolk S, Danz C. Buprenorphine versus methadone maintenance therapy: a randomized double-blind trial with 405 opioid-dependent patients. Addiction 2003; 98: 441-52.

© 2018 Fitzpatrick T et al. *JAMA Network Open.*

Extension Study Reference	Original Trial Reference
Huttunen JK, Heinonen OP, Manninen V, Koskinen P, Hakulinen T, Teppo L, et al. The Helsinki Heart Study: an 8.5-year safety and mortality follow-up. J Intern Med 1994; 235(1): 31-9.	Manninen V, Elo MO, Frick MH, Haapa K, Heinonen OP, Heinsalmi P, et al. Lipid Alterations and Decline in the Incidence of Coronary Heart Disease in the Helsinki Heart Study. JAMA 1988; 260(5): 641-51.
Lentine KL, Schnitzler MA, Xiao H, Brennan DC. Long-term safety and efficacy of antithymocyte globulin induction: use of integrated national registry data to achieve ten-year follow-up of 10-10 Study participants. Trials 2015; 16: 365.	Brennan DC, Daller JA, Lake KD, Cibrik D, Del Castillo D; Thymoglobulin Induction Study Group. Rabbit Antithymocyte Globulin versus Basiliximab in Renal Transplantation. N Engl J Med 2006; 355(19): 1967-77.
Levey AS, Greene T, Sarnak MJ, Wang X, Beck GJ, Kusek JW, et al. Effect of dietary protein restriction on the progression of kidney disease: long-term follow-up of the Modification of Diet in Renal Disease (MDRD) Study. Am J Kidney Dis 2006; 48(6): 879-88.	Klahr S, Levey AS, Beck GJ, Caggiula AW, Hunsicker L, Kusek JW, Striker G. The effects of dietary protein restriction and blood-pressure control on the progression of chronic renal disease. N Engl J Med 1994; 330: 877-84.
Lewinter C, Thygesen L, Doherty P, Køber L, Zwisler AD. Effectiveness of cardiac rehabilitation in HF patients: a long-term follow-up of the DANREHAB-trial. Eur J Heart Fail 2014; 16: 60.	Zwisler AD, Soja AM, Rasmussen S, Frederiksen M, Abedini S, Appel J, et al. Hospital-based comprehensive cardiac rehabilitation versus usual care among patients with congestive heart failure, ischemic heart disease, or high risk of ischemic heart disease: 12-month results of a randomized clinical trial. Am Heart J. 2008; 155(6): 1106-13.
Marcus PM, Bergstralh EJ, Fagerstrom RM, Williams DE, Fontana R, Taylor WF, Prorok PC. Lung cancer mortality in the Mayo Lung Project: Impact of extended follow-up. J Natl Cancer Inst 2000; 92(16): 1308-16.	Fontana RS, Sanderson DR, Taylor WF, Woolner LB, Miller WE, Muhm JR, Uhlenhopp MA. Early Lung Cancer Detection: Results of the Initial (Prevalence) Radiologic and Cytologic Screening in the Mayo Clinic Study. Am Rev Respir Dis 1984; 130:561-565.
McNamara HC, Wood R, Chalmers J, Marlow N, Norrie J, MacLennan G, et al. STOPPIT baby follow-up study: The effect of prophylactic progesterone in twin pregnancy on childhood outcome. PLoS One 2015; 10(4): e0122341.	Norman JE, Mackenzie F, Owen P, Mactier H, Hanretty K, Cooper S, et al. Progesterone for the prevention of preterm birth in twin pregnancy (STOPPIT): a randomised, double-blind, placebo-controlled study and meta-analysis. Lancet 2009; 373: 2034–40.
Miller AB, Wall C, Baines CJ, Sun P, To T, Narod SA. Twenty-five year follow-up for breast cancer incidence and mortality of the Canadian National Breast Screening Study: randomised screening trial. BMJ 2014; 348: g366.	Miller AB, Baines CJ, To T, Wall C. Canadian National Breast Screening Study: 2. Breast cancer detection and death rates among women aged 50 to 59 years. CMAJ 1992; 147 (10): 1477-88.
Myles PS, Peyton P, Silbert B, Hunt J, Rigg JR, Sessler DI; ANZCA Trials Group Investigators. Perioperative epidural analgesia for major abdominal surgery for cancer and recurrence-free survival: randomised trial. BMJ 2011; 342: d1491.	Rigg JR, Jamrozik K, Myles PS, Silbert BS, Peyton PJ, Parsons RW, et al. Epidural anaesthesia and analgesia and outcome of major surgery: a randomised trial. Lancet 2002; 359: 1276–82.
Puntoni M, Petrera M, Campora S, Garrone E, Defferrari C, Torrisi R, et al. Prognostic significance of VEGF after twenty year follow-up in a randomized trial of fenretinide in non-muscle invasive bladder cancer. Cancer Prev Res (Phila) 2016; 9(6):437-44.	Decensi A, Bruno S, Costantini M, Torrisi R, Curotto A, Gatteschi B, et al. Phase iia study of fenretinide in superficial bladder cancer, using DNA flow cytometry as an intermediate end point. J Natl Cancer Inst 1994; 86(2): 137-9.

© 2018 Fitzpatrick T et al. *JAMA Network Open.*

Extension Study Reference	Original Trial Reference
Sandblom G, Varenhorst E, Rosell J, Löfman O, Carlsson P. Randomised prostate cancer screening trial: 20-year follow-up. BMJ 2011; 342: d1539.	Pedersen KV, Carlsson P, Varenhorst E, Löfman O, Berglund K. Screening for carcinoma of the prostate by digital rectal examination in a randomly selected population. BMJ 1990; 300: 1041-4
Thomsen JL, Parner ET, Karlsmose B, Thulstrup AM, Lauritzen T, Engberg M. Secondary healthcare contracts after multiphasic preventive health screening: a randomized trial. Fam Pract 2005; 22(3): 242-8.	Lauritzen T, Leboeuf-Yde C, Lunde IM, Nielsen KD. Ebeltoft project: baseline data from a five-year randomized, controlled, prospective health promotion study in a Danish population. Br J Gen Pract 1995; 45: 542-7.
Virtamo J, Taylor PR, Kontto J, Männistö S, Utriainen M, Weinstein SJ, et al. Effects of alpha-tocopherol and beta-carotene supplementation on cancer incidence and mortality: 18-Year postintervention follow-up of the Alpha-Tocopherol, Beta-Carotene Cancer Prevention Study. Int J Cancer 2014, 135(1): 178085.	Virtamo J, Pietinen P, Huttunen JK, Korhonen P, Malila N, Virtanen MJ, et al. Incidence of Cancer and mortality following alpha-Tocopherol and Beta-Carotene Supplementation. JAMA 2003; 290(4): 476-85.
**Therapeutic benefits lost in the trial extension phase**	
Clayton PA, McDonald SP, Chapman JR, Chadban SJ. Mycophenolate vs azathioprine for kidney transplantation: 15-year follow-up of a randomized trial. Transplantation 2012; 94(2):152-8.	The Tricontinental Mycophenolate Mofetil Renal Transplantation Study Group. A blinded, randomized clinical trial of mycophenolate mofetil for the prevention of acute rejection in cadaveric renal transplantation. Transplantation 1996; 61(7): 1029-37.
Henderson RA, Jarvis C, Clayton T, Pocock SJ, Fox KA. 10-year mortality outcome of a routine invasive strategy versus a selective invasive strategy in non-ST-segment elevation acute coronary syndrome: The British Heart Foundation RITA-3 randomized trial. J Am Coll Cardiol 2015; 66(5): 511-20.	Fox KA, Poole-Wilson PA, Henderson RA, Clayton TC, Chamberlain DA, Shaw TR, et al. Interventional versus conservative treatment for patients with unstable angina or non-ST-elevation myocardial infarction: the British Heart Foundation RITA 3 randomised trial. Lancet 2002; 360: 743–51.
Ishani A, Grandits GA, Grimm RH, Svendsen KH, Collins AJ, Prineas RJ, Neaton JD. Association of single measurements of dipstick proteinuria, estimated glomerular filtration rate, and hematocrit with 25-year incidence of end-stage renal disease in the Multiple Risk Factor Intervention Trial. J Am Soc Nephrol 2006; 17(5): 1444-52.	Hafström L, Rudenstam CM, Blomquist E, Ingvar C, Jönsson PE, Lagerlöf B, et al. Regional Hyperthermic Perfusion with Melphalan After Surgery for Recurrent Malignant Melanoma of the Extremities. J Clin Oncol 1991; 9(12): 2091-4.
McArdle CS, McMillan DC, Greenlaw N, Morrison DS. Adjuvant radiotherapy and chemotherapy in breast cancer: 30 year follow-up of survival. BMC Cancer 2010; 10: 398.	McArdle CS, Crawford D, Dykes EH, Calman KC, Hole D, Russell AR, Smith DC. Adjuvant radiotherapy and chemotherapy in breast cancer. Br J Surg 1986; 73(4): 264-6.
Moss SM, Wale C, Smith R, Evans A, Cuckle H, Duffy SW. Effect of mammographic screening from age 40 years on breast cancer	Moss S. A trial to study the effect on breast cancer mortality of annual mammographic screening in women starting at age 40. J Med Screen 1999;6:144–148.

© 2018 Fitzpatrick T et al. *JAMA Network Open.*

Extension Study Reference	Original Trial Reference
mortality in the UK Age trial at 17 years' follow-up: A randomised controlled trial. Lancet Oncol 2015; 16(9): 1123-32.	
Roodnat JI, Hilbrands LB, Hené RJ, de Sévaux RG, Smak Gregoor PJ, Kal-van Gestel JA, et al. 15-year follow-up of a multicenter, randomized, calcineurin inhibitor withdrawal study in kidney transplantation. Transplantation 2014; 98(1): 47-53.	Smak Gregoor PJ, de Sévaux RG, Ligtenberg G, Hoitsma AJ, Hené RJ, Weimar W, et al. Withdrawal of Cyclosporine or Prednisone Six Months after Kidney Transplantation in Patients on Triple Drug Therapy: A Randomized, Prospective, Multicenter Study. J Am Soc Nephrol 2002; 13: 1365–73.
**Trial extension analyzed as observational study - i.e. not according to randomization**	
Bale G, Martínez-Camblor P, Burge PS, Soriano JB. Long-term mortality follow-up of the ISOLDE participants: causes of death during 13 years after trial completion. Respir Med 2008; 102(10): 1468-72.	Burge PS, Calverley PM, Jones PW, Spencer S, Anderson JA, Maslen TK. Randomised, double blind, placebo controlled study of fluticasone propionate in patients with moderate to severe chronic obstructive pulmonary disease: the ISOLDE trial. BMJ 2000; 320: 1297–303.
Cygankiewicz I, Gillespie J, Zareba W, Brown MW, Goldenberg I, Klein H, et al. Predictors of long-term mortality in Multicenter Automatic Defibrillator Implantation Trial II (MADIT II) patients with implantable cardioverter-defibrillators. Heart Rhythm 2009; 6(4): 468-73.	Moss AJ, Zareba W, Hall WJ, Klein H, Wilber DJ, Cannom DS, et al. Prophylactic implantation of a defibrillator in patients with myocardial infarction and reduced ejection fraction. N Engl J Med 2002; 346(12): 877-83.
Dalsgaard EM, Vestergaard M, Skriver MV, Borch-Johnsen K, Lauritzen T, Sandbaek A. Socioeconomic position and cardiovascular risk factors among people with screen-detected Type 2 DM: six-year follow-up of the ADDITION-Denmark trial. Prim Care Diabetes 2014; 8(4): 322-9.	Griffin SJ, Borch-Johnsen K, Davies MJ, Khunti K, Rutten GE, Sandbæk A, et al. Effect of early intensive multifactorial therapy on 5-year cardiovascular outcomes in individuals with type 2 diabetes detected by screening (ADDITION-Europe): a cluster-randomised trial. Lancet 2001; 378: 156-67.
Korpelainen R, Keinänen-Kiukaanniemi S, Nieminen P, Heikkinen J, Väänänen K, Korpelainen J. Long-term Outcomes of Exercise Follow-up of a Randomized Trial in Older Women with Osteopenia. Arch Intern Med 2010; 170(17): 1548-56.	Korpelainen R, Keinänen-Kiukaanniemi S, Heikkinen J, Väänänen K, Korpelainen J. Effect of Exercise on Extraskeletal Risk Factors for Hip Fractures in Elderly Women with Low BMD: A Population-Based Randomized Controlled Trial. J Bone Miner Res 2006; 21(5):772-9.
Kümler T, Gislason GH, Køber L, Torp-Pedersen C. Persistence of the prognostic importance of left ventricular systolic function and heart failure after myocardial infarction: 17-year follow-up of the TRACE register. Eur J Heart Fail 2010. 12(8): 805-11.	Køber L, Torp-Pedersen C. Clinical Characteristics and mortality of patients screened for entry into the Trandolapril Cardiac Evaluation (TRACE) study. Am J Cardiol 1995; 76: 1-5.
Nelson MR, Chowdhury EK, Doust J, Reid CM, Wing LM. Ten-year legacy effects of baseline blood pressure 'treatment naivety' in the Second Australian National Blood Pressure study. J Hypertens 2015; 33(11): 2331-7.	Wing LM, Reid CM, Ryan P, Beilin LJ, Brown MA, Jennings GL, et al. A Comparison of Outcomes with Angiotensin-Converting–Enzyme Inhibitors and Diuretics for Hypertension in the Elderly. N Engl J Med 2003; 348(7):583-92.

© 2018 Fitzpatrick T et al. *JAMA Network Open.*

Extension Study Reference	Original Trial Reference
Novak M, Björck L, Giang KW, Heden-Ståhl C, Wilhelmsen L, Rosengren A. Perceived stress and incidence of Type 2 diabetes: a 35-year follow-up study of middle-aged Swedish men. Diabet Med 2013; 30(1): e8-16.	Wilhelmsen L, Berglund G, Elmfeldt D, Tibblin G, Wedel H, Pennert K, et al. The multifactor primary prevention trial in Goteborg, Sweden. Eur Heart J 1986; 7: 279-88.
Regieli JJ, Jukema JW, Doevendans PA, Zwinderman AH, Kastelein JJ, Grobbee DE, van der Graaf Y. Paraoxonase variants relate to 10-year risk in coronary artery disease: impact of a high-density lipoprotein-bound antioxidant in secondary prevention. J Am Coll Cardiol 2009; 54(14): 1238-45.	Jukema JW, Bruschke AV, van Boven AJ, Reiber JH, Bal ET, Zwinderman AH, et al. Effects of Lipid Lowering by Pravastatin on Progression and Regression of Coronary Artery Disease in Symptomatic Men with Normal to Moderately Elevated Serum Cholesterol Levels. The Regression Growth Evaluation Statin Study (REGRESS). Circulation. 1995; 91(10): 2528-40
Sharma A, Apostolidou S, Burnell M, Campbell S, Habib M, Gentry-Maharaj A, et al. Risk of epithelial ovarian cancer in asymptomatic women with ultrasound-detected ovarian masses: a prospective cohort study within the UK collaborative trial of ovarian cancer screening (UKCTOCS). Ultrasound Obstet Gynecol 2012; 40(3): 338-44.	Menon U, Gentry-Maharaj A, Hallett R, Ryan A, Burnell M, Sharma A, et al. Sensitivity and specificity of multimodal and ultrasound screening for ovarian cancer, and stage distribution of detected cancers: results of the prevalence screen of the UK Collaborative Trial of Ovarian Cancer Screening (UKCTOCS). Lancet Oncol 2009; 10(4): 327–40.
Smelov V, Elfström KM, Johansson AL, Eklund C, Naucler P, Arnheim-Dahlström L, Dillner J. Long-term HPV type-specific risks of high-grade cervical intraepithelial lesions: A 14-year follow-up of a randomized primary HPV screening trial. Int J Cancer 2015; 136(5); 1171-80.	Elfgren K, Rylander E, Rådberg T, Strander B, Strand A, Paajanen K, et al. Colposcopic and histopathologic evaluation of women participating in population-based screening for human papillomavirus deoxyribonucleic acid persistence. Am J Obstet Gynecol 2005; 193: 650–7.
White MC, Tulsky JP, Menendez E, Goldenson J, Kawamura LM. Incidence of TB in inmates with latent TB infection: 5-Year follow-up. Am J Prev Med 2005; 29(4): 295-301.	White MC, Tulsky JP, Goldenson J, Portillo CJ, Kawamura M, Menendez E. Randomized controlled trial of interventions to improve follow-up for latent tuberculosis infection after release from jail. Arch Intern Med 2002; 162: 1044-50.

† Individual extension studies may have reported outcomes belonging to multiple categories so the numbers in this table differ from those in Table 3 (Summary of results of trial extension studies), which categorizes all reported analyses (n=155). In this table studies were placed in categories according to a hierarchy in which superiority in the trial extension phase> harms in the trial extension phase> null effects in the trial extension phase> benefits lost in the trial extension phase > analyzed as an observational study. Thus, each study only appears once in the table (n=113).

© 2018 Fitzpatrick T et al. *JAMA Network Open.*

eTable 4. Details of participants interventions and outcomes measured in the original and extended trials included in the review

Extension Study *	Original Trial*	Original Study Inclusion Criteria	Original Intervention	Original Trial Outcomes	Extended Trial Outcomes
**Statistically significant superiority of one treatment observed in trial extension phase**					
Aasa M, 2010.	Svensson L, 2006.	Men and women with ST elevation myocardial infarction. Patients >18 years old presenting within 6 hours after onset of symptoms, with a duration of z30 minutes and with ST-segment elevation >2 mm in at least 2 adjacent precordial leads or >1 mm in at least 2 adjacent limb leads were considered for inclusion. Recruited from 7 hospitals and their ambulance organizations	I: enoxaparin followed by abciximab and transfer to invasive center for optional PCI;<br>C: enoxaparin followed by reteplase;	Primary: ST-segment resolution 120 minutes and TIMI flow at coronary angiography 5 to 7 days after randomization.<br>Secondary: composite of death, stroke, or reinfarction	Primary: All cause mortality, cardiac death, myocardial infarction
Alehagen U, 2015.	Alehagen U, 2013.	All citizens of a rural Swedish city aged between 70 and 88 years who had previously participated in an epidemiological study and had been continuously followed with medical examinations since 1998. Patients were excluded for the following reasons: Recent myocardial infarction (within 4 weeks), Planned cardiovascular operative procedure within 4 weeks, Hesitation concerning whether the candidate can decide for him/herself to participate in the study or not, or if he/she understands the consequences of participation, Serious disease that substantially reduces survival or when it was not expected that the participant could	I: combination of 200 mg/day of coenzyme Q10 capsules and 200 µg/day of organic selenium yeast tablets;<br>C: placebo	Primary: Cardiovascular mortality;<br>Secondary: All cause mortality and cardiac function.	Primary: cardiovascular mortality;<br>Secondary: to establish if there were different effects on CV-morality in gender, diabetes, IHD and functional classes as measured by New York Heart Association functional Class (NYHA class) and mortality from other causes

© 2018 Fitzpatrick T et al. *JAMA Network Open.*

Extension Study *	Original Trial*	Original Study Inclusion Criteria	Original Intervention	Original Trial Outcomes	Extended Trial Outcomes
		cooperate for the full 4 year period and Other factors making participation unreasonable, such as long/complicated transport to the Primary Health Center where the project was managed, or drug/alcohol abuse.			
Arbel Y, 2016.	BIP Study, 2000.	Men and women 45 to 74 years of age, history of MI 6 months but ,5 years before enrollment into the study and/or stable angina pectoris confirmed by coronary angiography, and/or radionuclear studies or standard exercise tests. In addition, a lipid profile of serum total cholesterol between 180 to 250 mg/dL, LDL-C #180 mg/dL (#160 mg/dL for patients >50 years), HDL-C #45 mg/dL, and triglycerides #300 mg/dL was required.	I: 400 mg of bezafibrate per day;<br>C: placebo;<br>Co-I: dietary advice. Also, July 1994 onwards, Colestipol was given concomitantly with the study medication to 165 patients (57 patients in the bezafibrate and 107 in the placebo group) during the study.	Primary: fatal or nonfatal myocardial infarction or sudden death.<br>Secondary: included hospitalization for unstable angina, percutaneous transluminal coronary angioplasty, and coronary artery bypass grafting.	Fatal or non-fatal myocardial infarction or sudden death.
Arnsrud Godtman R, 2015.	Hugosson J, 2010.	Men born between 1930 and 1944 (age 50–64, median 56 years) living in the city of Göteborg, Sweden	I: screening group invited for Prostate Specific Antigen testing every 2 years;<br>C: not invited	Prostate-cancer specific mortality	Cumulative prostate cancer incidence and mortality
Aronson NE,	Aronson JD, 1946.	Indian children and young adults, living on different reservations and communities in various parts of the United States and Alaska, who were free from tuberculosis as indicated by their failure to react, definitely, to a maximum dose (0.005 mg.) of a standardized tuberculin PPD	I: vaccinated intracutaneously with freshly prepared BCG vaccine<br>C: sterile physiological salt solution	Efficacy of BCG vaccine	Efficacy of BCG vaccine

© 2018 Fitzpatrick T et al. *JAMA Network Open.*

Extension Study *	Original Trial*	Original Study Inclusion Criteria	Original Intervention	Original Trial Outcomes	Extended Trial Outcomes
Arriagada R, 2010.	Rutqvist LE, 1989.	Pre- and postmenopausal women aged below 71 years with a unilateral, operable breast cancer. Surgery consisted of a modified radical mastectomy. Patients were required to have either histologically verified lymph node metastases or a tumor diameter-measured on the surgical specimen- exceeding 30 mm.	I1: post-op Radiotherapy; I2: post-op Chemotherapy; C: surgery only	Recurrence free survival, overall survival	Loco-regional recurrence, distant metastases, recurrence free survival, overall survival, secondary malignancies
Austin J, 2008.	Austin J, 2005.	Men and women over the age of 60 years with heart failure, New York Heart Association (NYHA) class II or III, and left ventricular systolic dysfunction, confirmed by echocardiography, were eligible for the study.	c: 8-weekly monitoring of clinical status, optimization of pharmacotherapy, and self-care education in a specialist nurse–outpatient clinic (standard care, clinic based care).<br>i: in addition to standard care, twice-weekly 8-week multi-disciplinary cardiac rehab programme, including prescriptive exercise, individualized health education and information, and psychological and social support. (cardiac rehab)	Primary: functional status (NYHA class I–IV), functional performance (6 MWT), perceived exertion (Borg RPE), and health-related quality of life in terms of disease specific (MLHF) and cost utility (EuroQol) questionnaires.<br>Secondary: health care utilisation, in terms of the number and length of stay of hospital admissions arising from heart disease, and prescribed heart failure medication.	Primary: functional status, functional performance, and perceived exertion. HRQL measures, cost-utility questionnaires, EuroQol.<br>Secondary: healthcare use, routine biochemistry, and prescribed heart failure medication, current exercise history, all-cause mortality, survival.
Bjurstam N, 2003.	Bjurstam N, 1997.	The entire female population of the city of Gothenburg born between the years 1923 and 1944 inclusive. All women with a history of breast carcinoma prior to randomization were identified and excluded from	i: invitation for screening every 18 months<br>c: one screening around when the study group had their last screening	Primary: breast cancer mortality<br>Secondary: breast cancer incidence	Breast cancer mortality, breast cancer incidence, age-specific breast cancer mortality

© 2018 Fitzpatrick T et al. *JAMA Network Open.*

Extension Study *	Original Trial*	Original Study Inclusion Criteria	Original Intervention	Original Trial Outcomes	Extended Trial Outcomes
		the analysis, although not from the trial procedure.			
Bogner HR, 2007.	Bruce ML, 2004.	Men and women were recruited using a 2-stage sampling design. The study drew an age-stratified (60-74, 75 years), random sample of patients with an upcoming appointment. Physicians notified sampled patients by mail allowing patients to decline contact. Research associates telephoned the remaining sample to confirm study eligibility: age 60 years or older, ability to give informed consent, Mini-Mental State Examination (MMSE) score44 of 18 or higher, and ability to communicate in English. With oral consent, eligible patients were screened for depression using the Centers for Epidemiologic Studies Depression scale (CES-D). The study invited all patients with a CES-D score higher than 2046 as well as a 5% random sample of patients with lower scores to enroll in the research protocol.	I: Treatment guidelines tailored for the elderly with care management;<br>C: Usual care	Primary: Assessment of suicidal ideation and depression severity (Hamilton Scale)<br>Secondary: all cause and cause-specific mortality; disability (SF12 activities of daily living)	Mortality in patients with or without diabetes
Buchwald H, 2002.	Buchwald H, 1992..	Men and women between 30 and 64 years at randomization was the acceptable age range.<br>MI Study: Eligible patients had sustained one and only one electrocardiographically and enzymatically documented, atherosclerotic myocardial infarction (MI) 6-60 months prior to the date of	I: American Heart Association Phase II diet instruction and a partial illeal bypass operation.<br>C: American Heart Association Phase II diet instruction only	Primary: all-cause mortality<br>Secondary: death due to atherosclerosis and morbidity from recurrent myocardial infarction, unstable angina, cerebrovascular accident. Secondary:	Overall mortality

© 2018 Fitzpatrick T et al. *JAMA Network Open.*

Extension Study *	Original Trial*	Original Study Inclusion Criteria	Original Intervention	Original Trial Outcomes	Extended Trial Outcomes
		randomization. Patients with either a transmural or non-transmural MI were accepted.<br>Total plasma cholesterol study: To be eligible, the patient's total plasma cholesterol at the screening/baseline visit, after at least 6 weeks of dietary therapy, had to equal or exceed 220mg/dl, or if it was between 200 and 2 19 mg/dl, the concurrent LDL-cholesterol level had to equal or exceed 140 mg/dl.		coronary artery bypass surgery, percutaneous transluminal coronary angioplasty, cardiac transplantation, and peripheral vascular surgery, arteriographic changes as a predictor of subsequent clinical coronary events.<br>Combined end point of atherosclerotic coronary heart disease mortality or confirmed nonfatal myocardial infarction	
Danish Breast Cancer Cooperative Group, 2006.	Overgaard M, 1997.	Premenopausal high-risk patients with breast cancer. High-risk status was defined as consisting of one or more of the following: involvement of axillary lymph nodes, a tumor size of more than 5 cm, and invasion of the cancer to skin or pectoral fascia (pathological stage II or III). A woman was considered premenopausal if she had had amenorrhea for less than five years or had had a hysterectomy before the age of 55.	I: radiotherapy plus systemic chemotherapy; C: chemotherapy alone	Locoregional recurrence, overall survival, disease free survival	Locoregional recurrence and distant metastases
Davies WR, 2013.	Hoole SP, 2009.	1) Patients undergoing elective Percutaneous Coronary Intervention (PCI), 2) baseline cTnI below the lower limit of detection for the assay (<0.04 ng/mL), 3) 18 years of age or older 4) able to give informed consent 5) women could	I: remote ischemic pre-conditioning (induced by 3 5-minute inflations of a blood pressure cuff to 200 mm Hg around nondominated upper arm, followed by 5-minute	Primary: assess whether remote IPC given approximately 1 hour before elective PCI reduced cardiac troponin I (cTnI) concentration at 24	MACCE rate

© 2018 Fitzpatrick T et al. *JAMA Network Open.*

Extension Study *	Original Trial*	Original Study Inclusion Criteria	Original Intervention	Original Trial Outcomes	Extended Trial Outcomes
		not be of child bearing age 6) subjects could not take nicorandil (preconditioning mimetic) or glibenclamide (preconditioning blocking) 7) subjects have life expectancy of >6 months as assessed by their clinician.	intervals of reperfusions before arrival at the catheter lab).<br>Co-I: Percutaneous coronary intervention (performed via femoral arterial approach with 6F or 7F guiding catheters. All patients received aspirin, clopidogrel and other medications in accordance with local practice).<br>C: uninflated blood pressure cuff around the upper arm before arrival at the catheter lab.	hours.<br>Secondary: 1) effect of IPC on ischemic symptoms 2) ECG evidence of ischemia during coronary balloon occlusion, CRP, and major cardiac and cerebral events at 6 months.	
Dolan KA, 2005.	Dolan KA, 2003.	Male inmates were eligible to participate if they: (1) were assessed as suitable for MMT by a detailed interview with medical staff which confirmed they had a heroin problem; (2) were serving prison sentences longer than 4 months at time of interview; and (3) were able to provide signed informed consent.	i: Methadone maintenance<br>c: Wait list control	Heroin use, heroin injection and injection of other drugs, syringe sharing, HIV and HCV prevalence, tattooing and sexual risk behavior	Mortality, reincarceration, hepatitis C Seroconversion and HIV seroconversion.
Ekholm M, 2016.	Rydén L, 2005.	Pre-menopausal women with Stage 2 breast cancer (With and without ER and PR receptors) after breast sparing surgery and radiotherapy by Regional Oncological Centers	I: tamoxifen;<br>C: no treatment	Relapse free survival	All cause mortality and breast cancer related mortality
Flossmann E, 2007.	**UK TIA Study:** Farrell B, 1991.<br>**British Doctors**	**UK TIA:** men and women who had a recent transient ischaemic attack (TIA) or minor ischaemic stroke or had potential cardiac sources of embolism who were not anticoagulated. Exclusion: Patients	**UK TIA**:<br>I: 'High dose' aspirin (patients received two 300mg aspirin tablets twice daily);<br>co-I: 'Low dose' aspirin	**UK-TIA:**<br>Primary: survival free of disabling stroke, whilst definitely non-vascular deaths should be censored.	Primary: colorectal cancer.<br>Secondary: other cancers.

© 2018 Fitzpatrick T et al. *JAMA Network Open.*

Extension Study *	Original Trial*	Original Study Inclusion Criteria	Original Intervention	Original Trial Outcomes	Extended Trial Outcomes
	**Aspirin Study:** Peto R, 1988.	younger than 40 years of age, cerebrovascular event occurred before 3 months, previously experienced disabling major stroke, attacks were definitely due to something other than arterial thromboembolism, likely to experience adverse events from aspirin (allergy, intolerance, previous abnormal bleeding, alcoholism etc.) and confound analyses by taking aspirin 90 days prior to randomization or having myocardial infarction within 3 months prior to randomization, might have difficulty with follow-up, comply poorly and had severe intercurrent non vascular disease.<br>**BMDAS**: All male doctors resident in the United Kingdom in 1978 who were born this century, who had replied to a questionnaire about their smoking habits that was sent to them in 1951 (as part of another study), and who were still listed in the 1977 Medical Directory. Ineligible: already taking aspirin for various reasons, could not take it, and history of peptic ulcer, stroke, or definite myocardial infarction.	(patients received two 150 mg aspirin tablets in the morning and two placebo tablets in the evening);<br>C: Placebo (patients received two placebo tablets twice daily).<br>**BMDAS:**<br>I: daily aspirin (500mg) unless some contraindication was thought to have developed<br>C: avoid aspirin and products containing aspirin unless some specific indication for aspirin was thought to have developed (no placebo).	Secondary: time to "major stroke, myocardial infarction or vascular death".<br>**BMDAS**:<br>Primary: myocardial infarctions, strokes, and transient ischaemic attacks;<br>Secondary: deaths	
Folkesson J, 2005.	Pahlman L, 1993.	patients aged 79 and under with potentially resectable colorectal cancer (histopathologically proven)	I: reoperative irradiation (2.5 Gy inJive fractions over I week) followed by surgery within 1 week;<br>C: surgery alone	Postoperative (short term mortality) and operative complications	Primary: overall survival, cancer-specific survival.<br>Secondary: local recurrence rate, and distant metastasis

© 2018 Fitzpatrick T et al. *JAMA Network Open.*

Extension Study *	Original Trial*	Original Study Inclusion Criteria	Original Intervention	Original Trial Outcomes	Extended Trial Outcomes
Ford I, 2016.	Shepherd J, 1995.	Males aged 45-64 years who, at randomization, display at most minor overt evidence of CHD, identified from primary clinics throughout West of Scotland district. Men, who attended the first visit, whose non fasting plasma cholesterol was at least 252 mg/dL with no history of MI and LDL >155 mg/dL	I: pravastatin (40 mg each evening)<br>C: placebo	Primary: nonfatal myocardial infarction or death from coronary heart disease as a first event.<br>Secondary: death from coronary heart disease and nonfatal myocardial infarction.	Mortality, incident cancers, and cumulative number of hospital admissions
Frasure-Smith N, 1989.	Frasure-Smith N, 1985.	Male patients post myocardial infarction admitted to coronary care units in Montreal	I: Stress monitoring and psychological intervention after AMI;<br>C: usual care	Stress scores, rehospitalisations, LOHS and mortality	Recurrence of AMI and cardiac mortality
Fritz D, 2012.	de Gans J, 2002.	Men and women 17 years of age or older, had suspected meningitis in combination with cloudy cerebrospinal fluid, bacteria in cerebrospinal fluid on Gram's staining, or a cerebrospinal fluid leukocyte count of more than 1000 per cubic millimeter.	i: dexamethasone sodium phosphate (10 mg given every six hours iv for four days)<br>c: placebo	Primary: score on the Glasgow Outcome Scale<br>Secondary: death, focal neurologic abnormalities (defined as aphasia, cranial-nerve palsy, monoparesis, hemiparesis, and severe ataxia), hearing loss, gastrointestinal bleeding (clinically relevant bleeding with a decreased serum hemoglobin level), fungal infection, herpes zoster, and hyperglycemia (a blood glucose level higher than 144 mg per deciliter [8.0 mmol per	Survival

© 2018 Fitzpatrick T et al. *JAMA Network Open.*

Extension Study *	Original Trial*	Original Study Inclusion Criteria	Original Intervention	Original Trial Outcomes	Extended Trial Outcomes
				liter]). Audiologic examination was performed in patients with clinical hearing loss.	
Gitlin LN, 2009.	Gitlin LN, 2006.	Community-living adults (men and women) aged 70 and older who reported difficulty with one or more activities of daily living.	I: Multicomponent Home Intervention (five occupational therapy contacts (four 90-minute visits and one 20-minute telephone contact) and one physical therapy visit(90 minutes))<br>C: usual care	Instrumental activities of daily living	All cause mortality
Gjestad R, 2011.	Dahlgren L, 1989.	Patients were admitted mainly by self-referral. Only women in early phases of alcohol dependence are accepted. Signs of chronic alcoholism (liver cirrhosis, dementia), previous treatment for alcohol problems, and psychotic syndromes are regarded as exclusion criteria, as is use of narcotic drugs.	i: early treatment for women with alcohol addiction at specialized treatment center for women (EWA)<br>c: treatment as usual in regular ward at standard mixed-sex treatment facility (TAU) or the community	Primary and secondary outcomes not explicitly stated; assessed morbidity, mortality, alcohol consumption	All cause mortality
Goodin DS, 2012.	Duquette P, 1995.	Young men and women (mean age, 35.5 years) and ambulatory, with Kurtzke Expanded Disability Status Scale (EDSS) scores of 0 to 5.5 (mean, 2.9)	I1: 1.6 million international units (MIU) of interfero beta-1b every other day subQ;<br>I2: 8 MIU;<br>C: placebo	Primary: Annual exacerbation rates, annual change in EDSS and change in MRI lesion area;<br>Secondary: (1) annual exacerbation rates, (2) the proportion of exacerbation-free subjects, (3) the severity of exacerbation as measured by	Primary: survival/ mortality (all-cause mortality),<br>Secondary: all-cause mortality

© 2018 Fitzpatrick T et al. *JAMA Network Open.*

Extension Study *	Original Trial*	Original Study Inclusion Criteria	Original Intervention	Original Trial Outcomes	Extended Trial Outcomes
				quantitative changes in the NRS score (0 to 7 = mild, 8 to 15 = moderate, >15 = severe), and (4) activity and lesion burden found on the annual cranial MRI	
Hall AS, 1997.	AIRE Study Investigators, 1993.	Men and women 18 years of age or older admitted to coronary care, intensive care, or general medical units with a definite AMI and clinical evidence of heart failure at any time after the index AMI (usually sufficient to justify at least short-term diuretic or vasodilator treatment), were eligible.	I: ramipril on day 3 to day 10 after AMI (occurring on day 1)<br>c: placebo	Primary: total mortality.<br>Secondary: progression to heart failure, reinfarction, or stroke.	All-cause mortality
Hansen JM, 2008.	Wildner-Christensen M, 2003.	Men and women aged 40 to 64 (inclusive) years living in the city of Odense, Denmark, and the surrounding municipalities (rural and urban) were identified by their civil registration number in the county's population register.	I: offered Helicobacter pylori screening and eradication;<br>C: no invite	Primary: prevalence of dyspepsia;<br>Secondary: changes in consultation rate at the GP for dyspepsia, number of sick leave days because of dyspepsia, use of ulcer medication and antacids, number of diagnostic procedures for dyspepsia, and resulting diagnoses and quality of life.	Frequency of dyspepsia
Heart Protection Study Collaborative Group, 2011.	Collins R, 2003.	Men and women aged about 40–80 years with non-fasting blood total cholesterol concentrations of at least 3·5 mmol/L (135 mg/dL) were eligible provided they had a medical	I: 40 mg simvastatin daily;<br>C: placebo	First major coronary event (i.e., non-fatal myocardial infarction or coronary death) and of first major vascular	Primary: first post-randomisation major vascular event (defined as non-fatal myocardial infarction

© 2018 Fitzpatrick T et al. *JAMA Network Open.*

Extension Study *	Original Trial*	Original Study Inclusion Criteria	Original Intervention	Original Trial Outcomes	Extended Trial Outcomes
		history of: diabetes mellitus; coronary disease; occlusive disease of non-coronary arteries; or treated hypertension (if also male and aged at least 65 years).		event (i.e., major coronary event, stroke or revascularisation)	or coronary death, fatal or non-fatal stroke, coronary or non-coronary revascularisation). Secondary: major vascular events during each year of follow-up and in various subcategories of patients; major coronary events (i.e., nonfatal myocardial infarction or coronary death), strokes, and revascularisations separately; deaths from vascular and non-vascular causes separately; and cancers at all sites (excluding non-melanoma skin cancer).
Holm T, 1996.	Stockholm Rectal Cancer Study Group, 1990.	Men and women with biopsy confirmation of rectal adenocarcinoma, resectable tumors as judged by clinical examination, tumors not extending above the sacral promontory (lateral x-ray view), and no signs of distant metastases (physical examination, liver function tests, chest radiograph).	i: radiation therapy plus surgery<br>c: surgery alone	Primary: incidence of pelvic recurrence<br>Secondary: survival	Primary: adverse effects (venous thromboembolism, diseases of the arteries, femoral neck or pelvic fractures, intestinal obstructions, fistulas, diseases of

© 2018 Fitzpatrick T et al. *JAMA Network Open.*

Extension Study *	Original Trial*	Original Study Inclusion Criteria	Original Intervention	Original Trial Outcomes	Extended Trial Outcomes
					the genitourinary tract).<br>Secondary: mortality
Holme Ø, 2014.	Hoff G, 2009.	All residents aged 55-64 years living in the city of Oslo and Telemark County, Norway, who were registered and alive in the national population registry by November 1998 (n=55 736), were eligible	I: flexible sigmoidoscopy screening with or without a single round of faecal occult blood testing (once only);<br>C: no screening	Cumulative incidence and mortality of colorectal cancer after 5, 10, and 15 years.	Colorectal cancer incidence and mortality.
Hulse GK, 2003.	Hulse GK, 2002,	Men and women in psychiatric wards screened for excessive alcohol consumption within 18-65 years of age, given Alcohol Use Disorder Identification Test (AUDIT) - if they scored above 8, they were eligible for the study. Exclusion: had memory problems, apparent organic brain disease, lived outside the metropolitan area, required an interpreter, were insufficiently well to answer questions or were considered too aggressive to be interviewed	i: an information package which detailed safer alcohol consumption patterns and gave comparison scores for the alcohol consumption surveys.<br>i2: motivational interview which was an individual 45-minute treatment based on the non-judgemental approach	Weekly alcohol consumption in standard (10g) drinks (safe, hazardous, harmful)	Survival to first alcohol event (between intervention groups), hospital and mental health morbidity comparisons between intervention and matched groups
Jansen AF, 2009.	van Rooij J, 2003.	All successive patients (men and women) with corneal opacities attributed to HED who were scheduled for Penetrating Keratoplasty were considered for enrollment in this study.	I: Oral acyclovir 400 mg twice daily;<br>C: placebo tablets	Recurrence rate of herpetic eye disease–related events and rejection episodes, proven by viral cell culture or polymerase chain reaction.	Primary: Clinically evidence recurrence;<br>Secondary outcomes: visual acuity, graft failure, vascularization
Kim LG, 2007.	Ashton HA, 2002.	Men aged 65–74 years from four centres (Portsmouth, Southampton, Winchester, and Oxford) in the UK	i: invited for screening (for abdominal aortic aneurysm)	Primary: mortality related to abdominal aortic aneurysm	Primary: AAA-related mortality .

© 2018 Fitzpatrick T et al. *JAMA Network Open.*

Extension Study *	Original Trial*	Original Study Inclusion Criteria	Original Intervention	Original Trial Outcomes	Extended Trial Outcomes
		were identified from family doctor lists and Health Authority lists, after obtaining the family doctor's permission. Before randomisation, doctors were asked to list patients they considered unfit to be screened. These men were then excluded from the study.	c: no invitation for screening	Secondary: all cause mortality, frequency of ruptured abdominal aortic aneurysm, and effect of screening and surgery on quality of life	Secondary: all-cause mortality
Knatterud GL, 2000.	The Post Coronary Artery Bypass Graft Trial Investigators, 1997.	Men and women who were 21 to 74 years of age, had LDL cholesterol levels of no more than 200 mg per deciliter, and had had at least two saphenous-vein coronary bypass grafts placed 1 to 11 years before the start of the study. Of these patients, we deemed eligible those who had LDL cholesterol levels of 130 to 175 mg per deciliter (4.5 mmol per liter) and triglyceride levels below 300 mg per deciliter (3.4 mmol per liter), as measured at any visit to a study physician after the initiation of a Step 1 diet23; two patent saphenous-vein grafts (with stenosis of less than 75 percent) in men (one in women); and an ejection fraction of no less than 30 percent.	I1: aggressive-treatment group - lovastatin was initially given in doses of 40 mg per day.<br>i2: moderate treatment group - 2.5 mg per day lovastatin<br>co-I: warfarin or placebo (2x2 factorial design)	Primary: substantial progression of disease.<br>Secondary: grafts with occlusion, grafts with new lesions, grafts with substantial improvement, death, myocardial infarction, cancer, stroke, bypass surgery, angioplasty	All-cause mortality, myocardial infarction, revascularization, composite clinical outcome, other outcomes (stroke, peripheral vascular procedures, cancer).
Koh KK, 2016.	LIPID Study Group, 1998.	Men and women who had had an acute myocardial infarction or had a hospital-discharge diagnosis of unstable angina between 3 and 36 months before study entry. For patients to qualify for the study, the plasma total cholesterol level measured four weeks before	i: 40 mg of pravastatin once daily<br>c: matching placebo once daily.	Primary: death from coronary heart disease.<br>Secondary: death from any cause; death from cardiovascular causes; death from CHD or nonfatal myocardial infarction; myocardial	All-cause mortality, cause-specific mortality, cancer incidence

© 2018 Fitzpatrick T et al. *JAMA Network Open.*

Extension Study *	Original Trial*	Original Study Inclusion Criteria	Original Intervention	Original Trial Outcomes	Extended Trial Outcomes
		randomization was required to be 155 to 271 mg per deciliter and the fasting triglyceride level less than 445 mg per deciliter (5.0 mmol per liter).		infarction; stroke; non-hemorrhagic stroke; coronary revascularization (coronary angioplasty, coronary-artery bypass surgery, or both); number of days in the hospital; serum lipid levels; and the relation of changes in lipid levels to the occurrence of cardiovascular end points.	
Kostis JB, 2011.	SHEP Cooperative research group, 1991.	Men and women aged 60+ with SBP>160 and DBP<90, recruited using mass mailing and community screening techniques	I: Stepped treatment of isolated systolic hypertension: For step 1 of the trial, dose 1 was chlorthalidone, 12.5mg/d, or dose 2 was 25mg/d. For step 2, dose 1 was atenolol, 25mg/d, Dose 2 was 50mg/d;<br>C: placebo	Primary: Nonfatal and fatal (total) stroke. Secondary: Cardiovascular and coronary morbidity and mortality, all-cause mortality, and quality of life measures	Primary: CVD mortality. secondary: All cause mortality
Küchler T, 2007.	Kuchler T, 1999.	Male and female patients, recruited from surgical ward of a Hamburg hospital, having surgery for a range of gastrointestinal cancers: esophagus, stomach, liver/gallbladder, pancreas, or colon/rectum	I: psychotherapeutic support in addition to routine care<br>C: routine care in gastrointestinal cancers following surgery	Quality of life, overall survival	Overall survival
Labrie F, 2004.	Labrie F, 1999.	Men aged 45 to 80 years registered in the 1985 electoral rolls in metro Quebec City who were traceable in health registries. Exclusion: men with prostate cancer diagnosis prior	I: annual Prostate screening with PSA and DRE;<br>C: no screening invite letter	Death due to prostate cancer	Prostate cancer mortality

© 2018 Fitzpatrick T et al. *JAMA Network Open.*

Extension Study *	Original Trial*	Original Study Inclusion Criteria	Original Intervention	Original Trial Outcomes	Extended Trial Outcomes
		to Nov 1988 and men with prior screening/ referral to their clinic			
Lim WH, 2017.	Tedesco Silva H, 2010.	Men and women aged 18–70 years undergoing primary kidney transplantation were eligible. Key exclusion criteria included, kidneys donated after cardiac death or with a cold ischemia time >40 hours; donor age >65 years; recipients of multiorgan-, ABO-incompatible-, positive T-cell cross-match- or HLA-identical living-related-donor transplants; or most recent anti-HLA Class I panel-reactive antibodies (PRA) >20% by a CDC (complement-dependent cytotoxicity)-based assay or >50% by flow cytometry or ELISA.	I1: Everolimus 1.5 mg (0.75 mg p.o. b.i.d. targeted to 3–8 ng/mL) + RD-CsA.<br>i2: Everolimus 3.0 mg (1.50 mg p.o. b.i.d. targeted to 6–12 ng/mL) + RD-CsA.<br>C: MPA 1.44 g (720 mg p.o. b.i.d.) + ST-CsA.	Primary: composite efficacy failure (treated biopsy-proven acute rejection, graft loss, death or loss to follow-up).<br>Main safety endpoint: renal function (estimated glomerular filtration rate [eGFR], by Modification of Diet in Renal Disease [MDRD]) at Month 12 (last-observation-carried-forward analyses).	Primary: the first NMSC (i.e., basal cell carcinoma [BCC] or squamous cell carcinoma [SCC] of the skin) or any non-skin cancers diagnosed after transplantation.<br>Secondary: delayed graft function (defined as requirement for dialysis after transplantation); acute rejection; overall graft loss (defined as death or return to dialysis); death; eGFR at 1 and 6 months, 1, 2, 3, 5, and 7 years after transplantation (those who lost their grafts or died were censored); discontinuation rate (i.e., discontinued mycophenolic sodium in the MPA group or discontinued everolimus in the everolimus treatment groups),

© 2018 Fitzpatrick T et al. *JAMA Network Open.*

Extension Study *	Original Trial*	Original Study Inclusion Criteria	Original Intervention	Original Trial Outcomes	Extended Trial Outcomes
					adverse events (up to 2 years after transplantation, including infections, edema, hyperlipidemia, and wound complications); and composite endpoint of graft loss and death.
Lloyd SM, 2013.	Shepherd J, 2002.	Men and women aged 70–82 years were recruited if they had either pre-existing vascular disease (coronary, cerebral, or peripheral) or raised risk of such disease because of smoking, hypertension, or diabetes. Their plasma total cholesterol was required to be 4·0–9·0 mmol/L and their triglyceride concentrations less than 6·0 mmol/L.	I: pravastatin (40 mg per day);<br>C: placebo	Primary: a composite of coronary death, non-fatal myocardial infarction, and fatal or non-fatal stroke.<br>Secondary: examination of the coronary and cerebrovascular components separately.	Overall mortality, mortality from non-cardiovascular events, cardiovascular events, CHD and stroke
Lundström H, 2014.	de Fine Olivarius N, 2001.	All patients aged 40 or older with newly diagnosed diabetes between 1 March 1989 and 28 February 1991 based on hyperglycaemic symptoms or raised blood glucose values measured in general practice, or both, and who were registered with a participating general practitioner.	i: Regular follow up and individualized goal setting in the intervention group, follow up every three months and annual screening for diabetic complications were supported by sending a questionnaire to the general practitioner one month before the next expected consultation.<br>c: routine care (doctor	Primary: mortality, retinopathy, urinary albumin, myocardial infarction, stroke.<br>Secondary: peripheral neuropathy, angina pectoris, intermittent claudication, amputation	Any diabetes-related endpoint, diabetes-related deaths, all-cause mortality, myocardial infarction (MI), stroke, peripheral vascular disease and microvascular disease

© 2018 Fitzpatrick T et al. *JAMA Network Open.*

Extension Study *	Original Trial*	Original Study Inclusion Criteria	Original Intervention	Original Trial Outcomes	Extended Trial Outcomes
			decides their own course of action)		
Maas AC, 1999.	Simoons ML, 1985.	Men and women up to 70 years with severe chest pain lasting longer than 20 min with ST segment elevation of >0.2 mv in 1+ precordial leads and/or >0.1mV in 1+ limb leads. Also included patients with 0.2mV ST segment depression in precordial leads indicating posterior wall infarction.	i: treatment with intracoronary streptokinase OR treatment with intravenous + intracoronary streptokinase<br>c: conventional treatment - included Thalamonal and heparin followed by nicoumalone until hospital discharge	Mortality, ventricular fibrillation, pericarditis, cardiogenic shock, bleeding, reinfarction	Mortality/survival rates, reinfarction, subsequent cardiac events
Menon V, 2006.	Peterson JC, 1995.	Men and women aged 18 to 70. Serum creatine level of 1.2 to 7.0 mg/dL for women and 1.4 to 7.0 mg/dL for men or a creatinine clearance less than 70 mL/min; and mean arterial pressure of 125 mm Hg or less. Patients were excluded if they had diabetes requiring insulin, proteinuria of 10 g/d or more, or body weight less than 80% or more than 160% of standard body weight.	c: usual blood pressure goal<br>i: low blood pressure goal<br>STUDY A i: usual (1.3g/kg d) or low protein diet (0.58g/kg).<br>STUDY B: low or very-low (0.28g/kg) protein diet.	Rate of decline in glomerular filtration rate and change in proteinuria during follow-up.	All-cause mortality, cardiovascular mortality
Molyneux AJ, 2015.	Molyneux AJ, 2002.	Men and women were eligible for the trial if: (1) they had a definite subarachnoid haemorrhage, proven by computed tomography (CT) or lumbar puncture, within the preceding 28 days; (2) they had an intracranial aneurysm, demonstrated by intra-arterial or by CT angiography, which was considered to be responsible for the recent subarachnoid haemorrhage;	i: endovascular coiling<br>c: neurosurgical clipping	Primary: modified Rankin scale (number of patients dependent or dead after one year post treatment).<br>Secondary: prevention of rebleeding from the treated aneurysm, quality of life at 1 year (using a EuroQol measure), the	Death, dependence (measured according to patient-reported modified Rankin scale scores), and recurrent subarachnoid haemorrhage after neurosurgical

© 2018 Fitzpatrick T et al. *JAMA Network Open.*

Extension Study *	Original Trial*	Original Study Inclusion Criteria	Original Intervention	Original Trial Outcomes	Extended Trial Outcomes
		(3) they were in a clinical state that justified treatment, at some time, by either neurosurgical or endovascular means; (4) they had an intracranial aneurysm that was judged by both the neurosurgeon and the interventional neuroradiologist to be suitable for either technique on the basis of its angiographic anatomy; (5) there was uncertainty as to whether the ruptured aneurysm should be treated by neurosurgical or endovascular means; and (6) they gave appropriate informed consent, according to the criteria laid down by the local ethics committee.		frequency of epilepsy, cost-effectiveness, and neuropsychological outcomes.	clipping or endovascular coiling
Nielsen PH, 2011.	Andersen HR, 2003.	Men and women of 18 years or more, the presence of symptoms for at least 30 minutes but less than 12 hours, and cumulative ST-segment elevation of at least 4 mm in at least two contiguous leads.	i: primary angioplasty or fibrinolysis at invasive-treatment centers<br>c: primary angioplasty or fibrinolysis at referral hospitals	Composite of death from any cause, clinical reinfarction, disabling stroke at 30-days after treatment.	30 day mortality, long term (8-year) mortality
Nyström L, 2002.	**Malmo:** Andersson I, 1988.<br>**Two-Country**: Tabár L, 1985.<br>**Stockholm Trial**: Frisell J, 1986.<br>**Goteborg:** Bjurstam N, 1997.	**Malmo trial**: Women born between 1908 and 1932 (MMST **I**), women who were born 1933–45 and were living in Malmö between 1978 and 1990 (MMST **II**).<br>**Two County Trial**: Women aged 40–49, 50–69, and 70–74 years living in Kopparberg and Östergötland.<br>**Stockholm trial**: Women in the southern part of Stockholm born between 1917 and 1942.<br>**Göteborg trial**: All women born	**Malmo trial**: screen-film mammography alone, in the first two rounds with two views (craniocaudal and oblique) and in subsequent rounds with either two views or the oblique view alone depending on the parenchymal pattern.<br>**Two County Trial:** offered screening every 2 or 3 years depending on age	**Malmo trial**:<br>Primary: breast cancer as underlying cause of death.<br>**Two county trial**:<br>Primary: mortality from breast cancer.<br>**Stockholm trial**:<br>Primary: prevalence of breast cancer and metastasis.<br>**Goteborg trial:** breast carcinoma mortality	Breast cancer as underlying cause of death

© 2018 Fitzpatrick T et al. *JAMA Network Open.*

Extension Study *	Original Trial*	Original Study Inclusion Criteria	Original Intervention	Original Trial Outcomes	Extended Trial Outcomes
		between 1923 and 1944 who lived in the city of Göteborg.	(I), no screenings offered (C).<br>**Stockhom trial:** mammography alone with an oblique single-view scan (I), no screenings (C).<br>**Goteborg trial:** Two-view mammography was used unless the observations at the previous screen indicated that single-view mammography would be adequate, depending on the density of the breast (I), no screenings offered (C).		
Olds DL, 2014.	Kitzman H, 1997.	Women at less than 29 weeks' gestation, with no previous live births, and with at least 2 sociodemographic risk characteristics (unmarried, <12 years of education, unemployed).	I1: free transport for prenatal care<br>I2: free transport for prenatal care and developmental screening, referral services at ages 6, 12 and 24 months<br>I3: Free transport for prenatal care and nurse home visits during pregnancy and 2 postpartum<br>I4: I3 + home visits through child's 2nd birthday, as well as developmental screens and referrals	Primary: pregnancy induced hypertension, preterm delivery, low birth weight.<br>Secondary: children's injuries, ingestions and immunizations; mothers' reports of children's behavioural problems; tests of children's mental development; mothers' reports of subsequent pregnancy, educational achievement, and labor force participation; and use of welfare derived from state records	All-cause mortality in mothers and preventable-cause mortality in children (sudden infant death syndrome, unintentional injury, and homicide)

© 2018 Fitzpatrick T et al. *JAMA Network Open.*

Extension Study *	Original Trial*	Original Study Inclusion Criteria	Original Intervention	Original Trial Outcomes	Extended Trial Outcomes
Plüss CE, 2011.	Plüss CE, 2008.	Men and women (<75 years old) with an acute myocardial infarction or patients under-going coronary artery by-pass graft at Danderyd Hospital	I: recurrent case method learning dialogues at their primary healthcare centres: usual care plus stress management program, 5-day stay at patient hotel, cooking sessions with diet counselling;<br>C: usual rehab program: physical training, information/ counselling, heart school, outpatient clinic, individual counselling sessions<br>Co-I: all participants who smoked who offered a smoking cessation program	Primary: biochemical risk marker change (e.g. LDL cholesterol)<br>Secondary: exercise performance	Cardiovascular death, myocardial infarction, readmission for cardiovascular disease and days at hospital for cardiovascular reason
Puolakka K, 2004.	Möttönen T, 1999.	Men and women based on the criteria of the American Rheumatism Association for rheumatoid arthritis; age between 18 and 65 years; duration of symptoms of less than 2 years; active disease with three or more swollen joints and at least three of the following: erythrocyte sedimentation rate (ESR), at least 28 mm/h or C-reactive protein (CRP) concentration above 19 mg/L; morning stiffness of 29 min or more; more than five swollen joints and more than ten tender joints	I: Combination drug therapy (DMARDs (sulfasalazine, methotrexate, hydroxychloroquine) plus prednisolone);<br>C: Single drug therapy (DMARDs (sulfasalazine, methotrexate, hydroxychloroquine))	Primary: induction of remission (as per ACR criteria)<br>Secondary: meaningful clinical response (ACR 50% response criterion—i.e., minimum 50% improvement in tender and swollen joint plus a similar improvement in at least three of five remaining core measures); improvement in each individual core measure; development of radiographic joint	Cumulative duration of all sick leaves and RA-related disability pensions

© 2018 Fitzpatrick T et al. *JAMA Network Open.*

Extension Study *	Original Trial*	Original Study Inclusion Criteria	Original Intervention	Original Trial Outcomes	Extended Trial Outcomes
				damage; and frequency of adverse effects	
Qu C, 2014..	Sun Z, 1991.	All newborns in Qidong County of China (born 1985-90)	I: HBV vaccine;<br>C: no vaccine	Incidence of HBV infection, mortality/ adverse events from vaccination	Primary: mortality from development of hepatic encephalopathy and coagulopathy, PLC incidence;<br>Secondary: Brain tumour incidence
Rana MM, 2013.	The FUTURE II Study Group, 2007.	Women aged 15-26 years who were not pregnant, did not report abnormal results on a Papanicolaou smear, and had had a lifetime number of no more than four sex partners	I: Quadrivalent HPV vaccine (6/11/16/18), 3 doses;<br>C: placebo	Composite end point of cervical intraepithelial neoplasia grade 2 or 3, adenocarcinoma in situ, or cervical cancer related to HPV-16 or HPV-18	HPV16/18 associated cervical intraepithelial neoplasia (CIN) Grade 2/3 and invasive cervical carcinoma (ICC)
Rathsman B, 2013.	Reichard P, 1988.	Men or women with Type I Diabetes Mellitus (T1DM) aged 30 or less at onset with stipulated criteria in terms of non-proliferative retinopathy, normal renal function and unsatisfactory BG control	I: intensive control of T1DM<br>C: standard control of T1DM	Primary: HBA1c, retinopathy, microalbuminuria, nerve conduction studies, severe hypoglycemia	Foot ulceration and microcirculation
Ronco G, 2014.	**Swedescreen:** Forslund O, 2002.<br>**POBASCAM:** Bulkmans NW, 2004.<br>**ARTISTIC:** Kitchener HC, 2006.<br>**NTCC:**	**SWEDESCREEN**: women aged 32-38 participating in population-based screening program;<br>**POBASCAM**: women aged 30-60 years with a general practitioner;<br>**ARTISTIC**: women aged 20-64 years attending for routine cervical screening;<br>**NTCC**: women aged 25–60 years who were not pregnant, had never undergone hysterectomy, had not	**SWEDESCREEN:** HPV Screening + Cytology vs. Cytology only<br>**POBASCAM:** intervention based on cytology and hpv test, control group just based on conventional cytology and no hpv test.<br>**ARTISTIC:** cervical sample either concealed from or revealed to them	**SWEDESCREEN**: Primary: incidence of CIN2/CIN3+ lesions.<br>**POBASCAM**: Primary: proportion of histologically confirmed >CIN3 lesions in each study arm.<br>**ARTISTIC**: primary: proportion of additional lesions in the revealed	Primary: invasive carcinoma of the cervix.<br>Secondary: detection rate

© 2018 Fitzpatrick T et al. *JAMA Network Open.*

Extension Study *	Original Trial*	Original Study Inclusion Criteria	Original Intervention	Original Trial Outcomes	Extended Trial Outcomes
	Ronco G, 2010.	been treated for CIN in the last 5 years, and who were attending for a new routine cervical screening episode.	**NTCC**: intervention of conventional cytology vs. HPV test	arm, 3 year detection rate.<br>Secondary: sensitivity and specificity of test, cost effectiveness, psychological effects, attendance rates, test preferences.<br>**NTCC**: Primary: number of women with histologically confirmed pre-invasive lesions and invasive cervical cancer detected after randomisation.	
Rosell J, 2013.	Nordenskjöld B, 2005.	Postmenopausal women younger than 75 years of age who had early stage, axillary lymph node-negative or -positive, invasive disease	I: 5 years of adjuvant treatment with tamoxifen in breast cancer;<br>C: 2 years of treatment	Primary: event-free survival (local-regional recurrence, distant metastasis, contralateral breast cancer, or death) and overall survival.	Hospitalisation for non-fatal and fatal CHD and other forms of cardiac diseases, e.g., heart failure (HF), atrial fibrillation
Rousseau MF, 2016.	SOLVD Investigators, 1991.	Men and women with congestive heart failure, ejection fraction < 0.35 already taking drugs other than an angiotensin converting enzyme inhibitor as part of treatment for congestive heart failure.	i: enalapril at an initial dose of 2.5 mg twice daily, gradually increase to 10 mg twice daily unless side effects developed<br>c: placebo	Total mortality.<br>Secondary: incidence of heart failure, rate of heart failure	Primary: total mortality.<br>Secondary: cause-specific mortality
Scholefield JH, 2012.	Hardcastle JD, 1996.	Men and women aged 45–74 years who lived in the Nottingham area of the UK (from GP registry) without serious illness	I: FOB screening every 2 years<br>C: no screening	Colorectal cancer (CRC) mortality	CRC mortality
Schröder FH, 2014.	Schröder FH, 2009.	Men aged 50-74 at time of randomization. Sweden: 50-54; NED, Italy, Belgium, and Spain: up	i: screening for prostate cancer once every 4 years<br>c: no screening	Mortality from prostate cancer	Primary: Mortality due to prostate

© 2018 Fitzpatrick T et al. *JAMA Network Open.*

Extension Study *	Original Trial*	Original Study Inclusion Criteria	Original Intervention	Original Trial Outcomes	Extended Trial Outcomes
		to age 74; SWITZ: 55-69; Finland; 55, 59, 63 and 67.			cancer. Secondary: all-cause mortality
Secher RG, 2015.	Petersen L1, 2005.	Men and women aged 18-45 years with first episode of schizophrenia spectrum disorder who had not been given antipsychotic drugs for more than 12 continuous weeks; identified from all inpatient and outpatient mental health services in Copenhagen and Aarhus County	I: Integrated treatment (assertive community treatment with programmes for family involvement and social skills training);<br>C: Standard treatment (contact with a community mental health centre)	Primary: psychotic symptoms (hallucinations, delusions, and thought disorders) and negative symptoms (anhedonia, avolition, affective blunting, and alogia).<br>Secondary: substance abuse, treatment adherence, lower dosage of antipsychotic medication, higher satisfaction with treatment, and reduced burden to the family	Primary: psychotic symptoms (hallucinations, delusions, and thought disorders) and negative symptoms (anhedonia, avolition, affective blunting, and alogia).<br>Secondary: substance abuse, treatment adherence, lower dosage of antipsychotic medication, higher satisfaction with treatment, and reduced burden to the family
Sever PS, 2011.	Sever PS, 2005.	Men and women 40-79 years; Hypertension (either of: Untreated hypertension with sBP > 160mmHg or dBP > 100mmHg, or both OR Treated hypertension with sBP > 140mmHg or dBP > 90 mmHg, or both); Three or more cardiovascular risk factors, defined as:<br>Left ventricular hypertrophy (by ECHO or ECG), Type 2 diabetes, Peripheral arterial disease, Previous stroke or TIA, Male	I: combination antihypertensive regimen of a CCB (amlodipine) and ACEI (perindopril)<br>C: B-blocker (atenolol) and a thiazide diuretic (bendroflumethiazide).	Primary: nonfatal myocardial infarction, fatal coronary artery disease<br>Secondary: fatal and non-fatal strokes	Primary: all-cause mortality<br>Secondary: CV-related deaths, non-CV-related deaths, deaths from infection or respiratory illness

© 2018 Fitzpatrick T et al. *JAMA Network Open.*

Extension Study *	Original Trial*	Original Study Inclusion Criteria	Original Intervention	Original Trial Outcomes	Extended Trial Outcomes
		gender, Age >= 55 years, Microalbuminuria or proteinuria, Smoking, Ratio of plasma total cholesterol to HDL-cholesterol >=6, or Family history of premature CHD. Exclusions: prior MI, currently treated angina, Cerebrovascular event in past 3 months, fasting triglycerides >4.5 mmol/L, HF, uncontrolled arrythmia, clinically significant hematological or biochemical abnormality			
Stamler J, 2012.	Stamler J, 1996.	Men whose risk for coronary heart disease was assessed using measurements of serum cholesterol, diastolic BP, and self-reported cigarette smoking. Men who were in the upper 15% (changed to 10% after one third of the screening was completed) of risk, a serum creatinine 2.0 mg/dl and without evidence of cardiovascular or other life-threatening diseases were included.	I: special intervention (SI) received group and individual counseling on a fat-modified diet; a stepped-care drug treatment program for hypertension (after an initial attempt to control BP by weight reduction, if indicated); and for cigarette smokers, counseling aimed to achieve cessation.<br>C: usual care	Primary: mortality from coronary heart disease<br>Secondary: blood pressure	Primary: CVD Mortality.<br>Secondary: non-fatal CVD outcomes (CHD, MI, CHF, coronary surgery)
Stewart JC, 2014.	Unützer J, 2002.	Men and women were selected based on the following 2 strategies. The first strategy relied on referrals of depressed older adults from primary care practitioners, other clinic staff, or patients themselves in response to clinic promotions of the program. The second method consisted of systematic depression screening of English-speaking,	I: IMPACT intervention (access to a depression care manager who was supervised by a psychiatrist and a primary care expert and who offered education, care management, and support of antidepressant management by the	Depression, depression treatments, satisfaction with care, functional impairment, and quality of life	Primary: hard CVD event: a) fatal MI (ICD-10 codes I21-I22 the first-listed cause of death), (b) laboratory evidence of acute MI (creatine kinase-myocardial band isoenzyme value >3.0 ng/ml or

© 2018 Fitzpatrick T et al. *JAMA Network Open.*

Extension Study *	Original Trial*	Original Study Inclusion Criteria	Original Intervention	Original Trial Outcomes	Extended Trial Outcomes
		older adults who used the participating primary care clinics with a 2-item depression screener adapted from the PRIMEMD study. Exclusion: drinking problem, bipolar or psychosis, in psychiatric treatment, severe cognitive impairment, or at acute risk of suicide.	patient's primary care physician or a brief psychotherapy for depresssion, Problem Solving Treatment in Primary Care); C: usual care		troponin value >0.3 μg/L), (c) acute MI diagnosis, (d) fatal stroke, or (e)hemorrhagic or non-hemorrhagic stroke diagnosis. Secondary: fatal/nonfatal MI (categories a–c), fatal/nonfatal MI – cardiac enzyme confirmed (categories a and b), fatal/nonfatal stroke (categories d and e), and all-cause mortality
Stewart S, 2014.	Stewart S, 2012.	Men and women: 1) age 18 years or older; 2) discharged to home with a diagnosis of CHF as confirmed by a cardiologist; 3) persistent moderate-to-severe symptoms (New York Heart Association [NYHA] functional class II to IV); and 4) a recent history of 1 admissions for acute heart failure.	I: specialized CHF clinic-based intervention (CBI); C: home-based intervention (HBI)	Primary: all-cause, unplanned hospitalization or death during 12- to 18-month follow-up. Secondary: type and duration of hospitalization and healthcare costs.	Primary: event-free survival from all-cause emergency hospitalization or death, all-cause mortality; Secondary: rate of all-cause hospitalization and stay.
Strandberg TE, 2004.	4S Group, 1994.	Men and women aged 35–70 years with previous myocardial infarction or angina pectoris >6months prior, serum total cholesterol 5·5–8·0 mmol/L, and serum triglycerides 2·5 mmol/L or lower. Excluding:	i: simvastatin treatment c: placebo	Primary: all-cause mortality Secondary: major coronary event (coronary death, non-fatal acute MI,	Primary: All cause mortality, cardiovascular mortality, coronary mortality, cancer mortality.

© 2018 Fitzpatrick T et al. *JAMA Network Open.*

Extension Study *	Original Trial*	Original Study Inclusion Criteria	Original Intervention	Original Trial Outcomes	Extended Trial Outcomes
		premenopausal women of childbearing potential, secondary hypercholesterolaemia, unstable or Prinzmetal angina, tendon xanthomata, planned coronary artery surgery or angioplasty, MI during the preceding 6 months, antiarrhythmic therapy, congestive heart failure requiring treatment with digitalis, diuretics, or vasodilators, persistent atrial fibrillation, cardiomegaly, haemodynamically important valvular heart disease, history of completed stroke, impaired hepatic function, partial ileal bypass, history of drug or alcohol abuse, poor mental function, other serious disease, current treatment with another investigational drug, or hypersensitivity to HMG-CoA reductase inhibitors.		resuscitated cardiac arrest, silent MI. Tertiary: any coronary event, death or any atherosclerotic event, incidence of myocardial revascularization procedures, incidence of hospital admissions for acute CHD events	Secondary: Cancer incidence
Wallentin L, 2016.	FRISC II Investigators, 1999.	Men and women with symptoms of ischaemia that were increasing or occurring at rest, or that warranted the suspicion of acute myocardial infarction, with the last episode within 48 h before the start of dalteparin or standard heparin treatment. Exclusion criteria were raised risk of bleeding episodes, anaemia, or indication for or treatment in the past 24 h with thrombolysis, angioplasty in the past 6 months, being on a waiting list for coronary revascularisation,	I: Invasive treatments including coronary angiography within a few days of enrolment, aiming for revascularisation within 7 days of the start of open-label treatment. C: Non-invasive treatment included coronary angiography in patients with refractory or recurrent symptoms, despite maximum medical treatment, or severe	Primary: Mortality and myocardial infarction at 6 months of follow-up. Secondary outcomes: total death, myocardial infarction, symptoms of angina, need for late coronary angiography and revascularisation, bleeding episodes, and stroke.	Primary: composite of death or myocardial infarction. Secondary: all-cause death, cardiac death, and new revascularisation procedures. Additional composite endpoints, such as hospital admissions for ischaemic heart

© 2018 Fitzpatrick T et al. *JAMA Network Open.*

Extension Study *	Original Trial*	Original Study Inclusion Criteria	Original Intervention	Original Trial Outcomes	Extended Trial Outcomes
		other acute or severe cardiac disease, renal or hepatic insufficiency, known clinically relevant osteoporosis, other severe illness, hypersensitivity to randomised drugs, anticipated difficulties with cooperation or participation in this or another clinical trial. Patients with previous open-heart surgery, advanced age (eg, >75 years), or other disorders that made randomisation to early revascularisation inappropriate.	ischaemia on a symptom limited exercise test before discharge.<br>Half of the patients in each group were randomly assigned long-term treatment with subcutaneous dalteparin or placebo for 3 months.		disease and any cardiac disease.
**Harms observed in trial extension phase**					
Ajeganova S, 2014.	Svensson B1, 2005.	Men and women diagnosed as having RA according to the 1987 revised criteria of the American College of Rheumatology (formerly, the American Rheumatism Association), were ages 18–80 years, had a disease duration of 1 year, had active disease (defined by Disease Activity Score), and were started by the treating rheumatologist on the first DMARD	I: prednisolone 7.5 mg/d;<br>co-I: disease-modifying anti-rheumatic drug (DMARD);<br>C: DMARD alone	Primary: rheumatoid disease activity score and joint erosions;<br>Secondary: BMD	Composite CV event (ischaemic coronary and cerebrovascular events), components of the composite CV outcome, and death
Heliövaara-Peippo S, 2010.	Hurskainen R, 2001.	Women referred by general practitioners or gynaecologists to one of the five university hospitals in Finland because of menorrhagia, between November, 1994, and November, 1997.	I: levonorgestrel-releasing intrauterine system (IUS);<br>C: hysterectomy	Health related quality of life	Lower urinary tract symptoms, medications and operations for urinary incontinence
Hemminki E, 1995.	Hemminki E, 1991.	Mothers enrolled in the 27 participating maternity centers in Tampere and five neighboring	I: routine iron supplementation during pregnancy (100mg daily);	Fetal growth, increase in infections and subjective adverse	Next birth, perinatal mortality, proportions or

© 2018 Fitzpatrick T et al. *JAMA Network Open.*

Extension Study *	Original Trial*	Original Study Inclusion Criteria	Original Intervention	Original Trial Outcomes	Extended Trial Outcomes
		communities for the first time during a pregnancy were considered for inclusion in the study.	C: selective iron supplementation during pregnancy - if Hb fell below 100g/L after 14 wGA with lab-confirmed anemia, 50 mg ferrous sulfate was prescribed 2x daily for 2 months (or until Hb>110 g/L)	effects, and/or delays in birth	preterm and low birth weight infants, mean gestation length and birth weight.
Jones JM, 1989.	Details extracted from: Cuzick J, 1987.	Manchester Christie Trial: randomized 1461 female patients between 1949 and 1955. All patients were treated surgically by a Halsted radical mastectomy.	Two different regimens of kilovoltage radiotherapy were used, initially the so-called quadrate technique and later the peripheral technique I: immediate post-operative DXT after radical mastectomy<br>C: delayed treatment ("watched group"). Randomisation determined by even or odd dates of birth	The data available from a systematic review were limited to all cause mortality.	Deaths and causes of death
Killander F, 2014	Ryden S, 1992.	Postmenopausal (> 5 years after menopause) patients under 71 years of age, with stage I1 breast cancer were included in the trial after modified radical mastectomy; specifically, patients had invasive mammary adenocarcinoma, and T1N+ or T2N0/N+, e.g. stage II with the tumour-node-metastasis (TNM) classification of that time.	I1: radiotherapy (RT),<br>i2: radiotherapy and tamoxifen (RTAM) or<br>i3: tamoxifen without radiotherapy<br>(TAM).	Recurrences, contralateral breast cancer, new primary cancer, cause-specific mortality, all-cause mortality	Overall survival, time to breast cancer death, and fatal and non-fatal late side-effects of radiation.
Mortensen FV, 2011.	Jensen LS, 1996.	All patients admitted for elective colorectal surgery between January 1992 and January 1995 were evaluated for inclusion in the study.	I: buffy-coat poor red cells;<br>C: filtered leukocyte depleted red cells	Primary: postoperative wound infection.<br>Secondary: Non-surgical infections,	Primary: cause-specific mortality;<br>Secondary: cause-specific mortality:

© 2018 Fitzpatrick T et al. *JAMA Network Open.*

Extension Study *	Original Trial*	Original Study Inclusion Criteria	Original Intervention	Original Trial Outcomes	Extended Trial Outcomes
		Exclusion criteria were age less than 18 years, the need for emergency surgery, and the use of immunosuppressive drugs, except steroids.		anastomatic leakage, re-operation and death.	rectal cancer and CVD
Onsrud M, 2012.	Aalders J, 1980.	Women referred to the Norwegian Radium Hospital for primary and/or secondary treatment of stage 1 endometrial carcinoma of the corpus uteri; FIGO stage I cases were excluded	I: Vaginal brachytherapy + external pelvic irradiation<br>C: brachytherapy alone	Primary: Local and remote recurrence of endometrial carcinoma<br>Secondary: survival	Survival, local and distant recurrence and secondary cancers
Perez MV, 2012.	Rossouw JE, 2002.	Postmenopausal women between the ages of 50 and 79 were recruited between 1993 and 1998 at 40 US clinical centers.	I1: women who had not had a hysterectomy were randomized to 0.625 mg/d of conjugated equine estrogens (CEE) plus 2.5 mg/d of medroxyprogesterone acetate (MPA) or placebo<br>i2: women with prior hysterectomy were randomized to 0.625 mg/d of CEE or placebo.	Primary: coronary heart disease (including non-fatal MI and death)<br>Secondary: cancer, fractures	Incident atrial fibrillation
Persson PE, 2005.	Persson PE, 1998.	Men and women operated on with primary THA for arthrosis	I1: Ibuprofen from the day of surgery for 3 weeks;<br>i2: Ibuprofen for 1 week and then placebo for 2 weeks;<br>c: Placebo for 3 weeks.	Primary: Functional grading according to Charnley, the Harris Hip Score and range of motion of the affected joint.<br>Secondary: Pre- and postoperative complications, type of anesthesia, blood loss and blood transfusions.	Primary: revisioning, Secondary: radiographic loosening
Radford L, 2011.	Reid IR, 2006.	Men and women aged more than 55 years, not receiving therapy for	i: 1 g of elemental calcium daily (as citrate).	Primary: time to first clinical fracture at any	Primary: fracture incidence.

© 2018 Fitzpatrick T et al. *JAMA Network Open.*

Extension Study *	Original Trial*	Original Study Inclusion Criteria	Original Intervention	Original Trial Outcomes	Extended Trial Outcomes
		osteoporosis or taking calcium supplements, and free of major ongoing disease. Serum creatinine was less than 2.3 mg/dL (0.2 mmol/L), and serum 25-hydroxyvitamin D was greater than 10 g/L (25 nmol/ L). Lumbar spine density was not below the age-appropriate normal range.	C: identical placebo in 2 divided doses.	site.<br>Secondary: bone density and the following fracture subgroups: total vertebral fractures, hip fractures, distal forearm fractures, and osteoporotic fractures (comprising all fractures except those of the head, hands, feet, and ankles, and resulting from major trauma).	Secondary: bone mineral density, cardiovascular events (MI, stroke, death)
Vessey MP, 1983.	Swyer GIM, 1954.	Women with their first pregnancy, attending University College hospital	I: stilboestrol varying dose (in total around 11g)<br>C: placebo	Late pregnancy toxaemia and perinatal mortality	Deaths, malignant and non-malignant tumors (mothers) plus a range of urogenital obstetric outcomes in female and male offspring
von Bormann B, 2015.	Busch OR, 1993.	Men and women scheduled for a potentially curative resection of cancer of the colon or rectum, did not have severe cardiovascular or respiratory disease, no history of epilepsy after infancy and a hemoglobin concentration above 11.3 g per deciliter. In addition, patients had to have no evidence of metastatic disease , no other cancer except basal cell carcinoma of the skin or in situ carcinoma of the cervix. No history of blood	I: allogeneic transfusion;<br>C: autologous-transfusion	Primary: Colorectal cancer-specific survival rates<br>Secondary: colorectal recurrence	Overall and Colorectal-cancer specific survival

© 2018 Fitzpatrick T et al. *JAMA Network Open.*

Extension Study *	Original Trial*	Original Study Inclusion Criteria	Original Intervention	Original Trial Outcomes	Extended Trial Outcomes
		transfusion during the three months before randomization.			
Winkel P, 2015.	Jespersen CM, 2000.	Men and women aged 18 to 85 years who had a discharge diagnosis of myocardial infarction or angina pectoris in 1993-9 and alive in August 1999 were invited by letter. Exclusion criteria included myocardial infarction or unstable angina within the previous three months; percutaneous transluminal coronary angioplasty or coronary bypass surgery within the previous six months; New York Heart Association class IV cardiac failure; impaired renal or hepatic function; active malignancy; intolerance to macrolides; treatment with methylxanthines, carbamazepine, cisapride, astemizole, terfenadine, or coumarin anticoagulants; participation in other clinical trials within the previous month; incapacity to manage own affairs or sign informed consent; breast feeding; and possible pregnancy.	I: oral clarithromycin 500 mg/day;<br>C: placebo treatment	Primary: composite of all cause mortality, myocardial infarction, or unstable angina pectoris during three years' follow-up.<br>Secondary: composite of cardiovascular mortality, myocardial infarction, or unstable angina pectoris.	Primary: All cause mortality, acute myocardial infarction, unstable angina pectoris;<br>Secondary: myocardial mortality, acute myocardial infarction, unstable angina pectoris
**Null effects in original trial also seen in the trial extension phase**					
Appel JM, 2012.	Ejlertsen B, 2007.	Women who had completely resected unilateral invasive carcinoma of the breast and no signs of distant metastases as determined by physical examination, chest radiography, and bone scintigraphy (if positive, to	I1: CMF (cyclophosphamide 600 mg/m2, methotrexate 40 mg/m2 and 5-fluorouracil 600 mg/ m2)<br>i2: CEF (cyclophosphamide 600	Primary: Symptoms, side effects, and findings on clinical examination, vital status.<br>Secondary: Haemoglobin, white	Survival, incidence of CHF, cardiac impairment

© 2018 Fitzpatrick T et al. *JAMA Network Open.*

Extension Study *	Original Trial*	Original Study Inclusion Criteria	Original Intervention	Original Trial Outcomes	Extended Trial Outcomes
		be confirmed by radiography), or axial bone radiography. Lower axillary clearance (level I and part of level II) in combination with breast-conserving surgery or mastectomy was required.	mg/m2, epirubicin 60 mg/m2 and 5-fluorouracil 600 mg/m2)	blood cell count, and platelet count.	
Beebe GW, 1972.	Beebe GW, 1964.	Not clearly stated - American army recruits	i: adjuvant influenza virus vaccine<br>c1: standard aqueous vaccine<br>c2: saline	Primary: specific causes of mortality; Secondary: morbidity, hospitalizations for various causes (e.g. allergy, cancer)	Mortality (cause-specific, as per ICD-7 classification)
Brouwers FP, 2011.	Asselbergs FW, 2004.	All residents of Groningen, Netherlands aged 28-75 were sent a questionnaire and asked to send in a morning urine sample. The key entry criteria of the PREVEND IT were persistent microalbuminuria (a urinary albumin concentration 10 mg/L in 1 early morning spot urine sample and a concentration of 15 to 300 mg/24 hours in 2 24-hour urine samples at least once), a blood pressure 160/100 mm Hg and no use of antihypertensive medication, and a total cholesterol level 8.0 mmol/L, or 5.0 mmol/L in case of previous yocardial infarction, and no use of lipid-lowering medication. The cutoff values for blood pressure and cholesterol were based on guidelines of general practitioners from the Netherlands in 1998. Important exclusion criteria were any of the following: creatinine clearance 60% of the normal age	I1: fosinopril 20 mg;<br>I2: pravastatin 40 mg;<br>C: matching placebo	Cardiovascular mortality and hospitalization for cardiovascular morbidity	Combined incidence of cardiovascular mortality and hospitalization for cardiovascular morbidity

© 2018 Fitzpatrick T et al. *JAMA Network Open.*

Extension Study *	Original Trial*	Original Study Inclusion Criteria	Original Intervention	Original Trial Outcomes	Extended Trial Outcomes
		adjusted value and use of ACE inhibitors or angiotensin II receptor antagonists.			
Cherry N, 2014.	Cherry N, 2002.	Women, age 50–69 years, admitted to coronary care units or general medicine wards in participating hospitals, who had survived a first myocardial infarction and were discharged within 31 days of admission. Individuals were recruited from 35 hospitals in England and Wales. Exclusion: previous MI, use of HRT, vaginal bleeding in past 12 months, history of breast/ ovarian/ endometrial cancer, active thrombophelitis or history of DVT, pulmonary embolism, acute or chronic liver disease, Rotor syndrome, Dubin-Johnson syndrome, or severe renal disease.	I: one tablet of oestradiol valerate (2 mg) daily;<br>C: placebo daily	Primary: reinfarction or cardiac death, and all-cause mortality.<br>Secondary: uterine bleeding, endometrial cancer, stroke or other embolic events, and fractures.	Death (all-cause, cardiac disease, stroke or cancer) and cancer incidence (any, breast or endometrium).
Cook NR, 2016.	The Trials of Hypertension Prevention Collaborative Research Group, 1992.	Healthy men and women, aged 30 through 54 years, who had high normal DBP and were not taking antihypertensive drugs for the prior 2 months. The BP eligibility level was based on three measurements at each of three screening visits (SV), between 10 and 30 days apart, with inclusion limits based on cumulative averages of all readings through that visit, as follows: SVI, 75 through 97 mm Hg; SV2, 77 through 94 mm Hg; and SV3 (baseline), 80 through 89 mm Hg.	I1: Life-style interventions (3 groups: Weight reduction, Sodium reduction and Stress management).<br>I2: Nutiritional supplements (2 groups: Calcium and Magnesium, and Fish Oil and Potassium after wash-out period).<br>C: Usual care for Life-style interventions and placebo for Nutritional supplements.	Primary: change in diastolic blood pressure from baseline to final follow-up, measured by blinded observers.<br>Secondary: changes in systolic blood pressure and intervention compliance measures.	Long term mortality

© 2018 Fitzpatrick T et al. *JAMA Network Open.*

Extension Study *	Original Trial*	Original Study Inclusion Criteria	Original Intervention	Original Trial Outcomes	Extended Trial Outcomes
Cushman WC, 2012.	ALLHAT Collaborative Research Group, 2003.	Men and women 55 years and older with untreated or briefly treated (less than 2 months) high blood pressure - systolic BP 140-180mmHg and/or diastolic BP 90-110 mmHg OR men/women 55+ who took less than 3 antihypertensive medications for at least 2 months with BP of 160/100mmHg. Inclusion also required a risk factor for CHD: prior CVD event (MI, Stroke), left ventricular hypertropy, history of T2DM, current cigarette smoker, and low HDL.	i: initial treatment of high-risk hypertension with calcium channel blocker (amlodipine), ACE inhibitor (lisinopril) or alpha-blocker (doxazosin)<br>c: standard treatment with chlorthalidone	Primary: fatal coronary heart disease, nonfatal myocardial infarction.<br>Secondary: all-cause and cause-specific mortality, stroke, heart failure, cardiovascular disease, end-stage renal disease	Primary: cardiovascular mortality.<br>Secondary: mortality, stroke, CHD, HF, end-stage renal disease
Delaney EK, 2008.	Campbell N, 1998.	Men and women with a history of coronary heart disease or prescribed nitrates, were <80 years old and recruited from GP practices	I: nurse-led secondary prevention clinics which promoted medical and lifestyle aspects of prevention and offered regular follow-up for 1-year;<br>C: usual care	Use of aspirin, BP management, Lipid Management, Smoking	All cause mortality and coronary events
Gao YC, 2017.	Rogers SJ, 2010.	Men and women aged 18 and older, classic biliary-type pain, at least 1 episode within the past 6 months, ultrasonographic demonstration of cholecystolithiasis, platelet count 100000, American Society of Aneseshesiologists risk grade 1 or 2.	i: laparoscopic cholecystectomy plus laparoscopic common bile duct exploration (LC LCBDE)<br>i2: endoscopic retrograde cholangiopancreatography sphincterotomy plus laparoscopic cholecystectomy (ERCP/S LC).	Primary: efficacy of stone clearance from common bile duct.<br>Secondary: length of hospital stay, cost of index hospitalization, professional fees, hospital charges, morbidity and mortality, and patient acceptance and quality of life scores.	Recurrent common bile duct stone disease or biliary complications

Extension Study *	Original Trial*	Original Study Inclusion Criteria	Original Intervention	Original Trial Outcomes	Extended Trial Outcomes
Gårdmark T, 2007.	Lundholm C, 1996.	Men and women with primary dysplasia, or stage Tis, stage T1, grade 3 and multiple recurrent stage TaPT1, grade 1 or 2 disease with normal liver and kidney function tests, and no chemotherapy during the prior 6 months. Exclusion criteria were previous or ongoing intravesical treatment with mitomycin C, BCG or radiotherapy, any secondary malignancy except treated carcinoma in situ of the uterine cervix or basal cell carcinoma of the skin, ongoing corticosteroid therapy, leukocytes less than 3,000/ mm., thrombocytes less than 100,000/mm., untreated urinary tract infection, urethral stricture preventing cystoscopy, active tuberculosis, pregnancy and expected difficulties during follow-up (for example Karnofsky performance index less than 50, senility, psychotic disease or any other reason that might prevent follow-up.	I: Mitomycin C (40 mg);<br>C: Pasteur strain BCG (120 mg)	Recurrence and progression of superficial bladder carcinoma.	Progression, later treatments and survival in this group of patients with high-risk urinary bladder cancer that was not muscle-invasive.
Gibson A, 2008.	Mattick RP, 2003.	Men and women with a current diagnosis of opioid dependence using the criteria in the fourth edition of the *Diagnostic and Statistical Manual of Mental Disorders* (American Psychiatric Association 1994); were aged 18 years or older; lived within commuting distance of the clinic; appeared mentally competent to	Patients received buprenorphine or methadone AND either placebo syrup or placebo tablet, as indicated clinically using a flexible dosage regime. During weeks 1–6, patients were dosed daily. From weeks 7–13, buprenorphine	Primary: Retention in treatment, and illicit opioid use as determined by urinalysis.<br>Secondary: Self-reported drug use, psychological functioning, HIV risk behavior, general	Primary: all cause mortality.<br>Secondary: survival and cause-specific mortality

© 2018 Fitzpatrick T et al. *JAMA Network Open.*

Extension Study *	Original Trial*	Original Study Inclusion Criteria	Original Intervention	Original Trial Outcomes	Extended Trial Outcomes
		give informed consent; and signed informed consent.	patients received double their week 6 dose on alternate days.	health and subjective ratings.	
Huttunen JK, 1994.	Manninen V, 1988.	The screening for participation was conducted in three steps within a period of three to five months. Cholesterol-lowering dietary counseling started three to six weeks prior to the drug treatment. Subjects free of coronary heart disease and other major disability were accepted, including those with mild hypertension and non-insulin-dependent diabetes. At the third pretreatment (baseline) visit, subjects meeting these acceptance criteria who expressed their willingness to participate were randomly allocated either to the gemfibrozil (n = 2046) or to the placebo (n = 2035) group.	I: gemfibrozil therapy 600mg twice daily;<br>C: placebo	Primary: incidence of heart disease (CHD);<br>Secondary: serum cholesterol level, HDL level and LDL level	Gastrointestinal symptoms, surgery, strokes, cancer incidence, mortality by cause.
Lentine KL, 2015.	Brennan DC, 2006.	Only adult candidates for renal transplants from deceased donors were considered for enrollment. Eligibility was determined according to the duration of cold ischemia and other donor and recipient risk factors. One or more of these factors, which put the recipient at high risk for acute rejection or delayed graft function, were required for eligibility. Patients were excluded if they had been receiving immunosuppressive therapy before transplantation; had received an investigational medication within the	I: rabbit antithymocyte globulin (1.5 mg per kilogram of body weight daily) during transplantation (day 0) and on days 1 through 4. Patients assigned to this group were given acetaminophen and diphenhydramine before receiving antithymocyte globulin (1.5 mg per kilogram of body weight given intravenously). Treatment with	Primary: efficacy end point, defined as a composite of the first occurrence of biopsy-proven acute rejection, delayed graft function, graft loss, or death; the incidence of each of these end points was also studied. All episodes of acute rejection were confirmed by biopsy, with histologic characteristics	Primary efficacy endpoint: FDA specified composite triple endpoint of allograft rejection, graft failure, or patient death used in clinical trials of immunosuppression efficacy.<br>Secondary: acute rejection, death-censored graft failure, and all-cause graft loss, evaluated

© 2018 Fitzpatrick T et al. *JAMA Network Open.*

Extension Study *	Original Trial*	Original Study Inclusion Criteria	Original Intervention	Original Trial Outcomes	Extended Trial Outcomes
		past 30 days; had a known contraindication to the administration of antithymocyte globulin or basiliximab; were suspected or known to have an infection or were seropositive for hepatitis B surface antigen (HBsAg), antibody against hepatitis B core antigen (anti-HBcAg), hepatitis C virus (HCV), or human immunodeficiency virus (HIV); or had had cancer (except nonmelanoma skin cancer) within the previous 2 years. Pregnant women, nursing mothers, and women of childbearing potential who were not using condoms or oral contraceptives were excluded.	antithymocyte globulin was initiated intraoperatively, before graft reperfusion. Subsequent doses were given daily through day 4, for a total dose of 7.5 mg per kilogram.<br>C: basiliximab (20 mg) on days 0 and 4. Basiliximab was given intravenously. Both groups received maintenance immunosuppressive therapy involving cyclosporine (modified), mycophenolate mofetil, and prednisone.	described according to the Banff criteria with the use of microscopy. Delayed graft function was<br>defined as the need for dialysis within the first week after transplantation. Slow graft function was defined as a serum creatinine level exceeding 3.0 mg per deciliter on day 5 that did not require treatment with dialysis.	individually at 10 years post-transplant.
Levey AS, 2006.	Klahr S, 1994.	Men and women age 18 to 70 years, a serum creatinine concentration of 1.2 to 7.0 mg/dL in women and 1.4 to 7.0 mg/dL in men or a creatinine clearance of less than 70 ml per minute per 1.73 m^2 of body surface area and a mean arterial pressure 125 mm Hg or less.	I: Low protein diet<br>C1: Usual protein diet; these groups were further compared between usual or low blood pressure [Study 1];<br>C2: Low protein diet; these groups were further compared between usual or low blood pressure [Study 2]	Rate of change in glomerular filtration rate	Kidney failure, defined as the requirement for dialysis therapy or kidney transplantation, and a composite of kidney failure and all-cause mortality.
Lewinter C, 2014.	Zwisler AD, 2008.	Men and women with CHF, IHD, or HR (high risk of heart disease) who were admitted to the Department of Cardiology of Bispebjerg Hospital. Exclusion criteria: severe or terminal illness, mental or social problems, transfer to another	I: Cardiac rehabilitation<br>C: Usual Care in cardiac patients	Primary: composite of total mortality, myocardial infarction, or acute first-time readmission due to heart disease.<br>Secondary:	Composite of all cause death, MI and acute hospital admission due to cardiac causes.

© 2018 Fitzpatrick T et al. *JAMA Network Open.*

Extension Study *	Original Trial*	Original Study Inclusion Criteria	Original Intervention	Original Trial Outcomes	Extended Trial Outcomes
		center, transfer to invasive center, living in nursing home, not speaking Danish, and other (not specified).		hospitalization, risk profile, and quality of life	
Marcus PM, 2000.	Fontana RS, 1984.	Male outpatients who are at high risk of lung cancer and who met the requirements of the prevalence screening. Participants were excluded if it appeared that the life expectancy was less than 5 years or that pulmonary resection would not be tolerated. Other exclusion criteria include a history of cancer of the respiratory tract, established diagnosis of lung cancer, evidence that either the patient or the referring physician suspected the presence of lung cancer at the time the patient entered the Mayo clinic.	I: patient was part of a close surveillance group, offered roentgenographic and cytologic rescreening every 4 months; Every candidate accepted for prevalence screening was scheduled for chest roentgenography and received assistance in completing a questionnaire. He was given instructions for collecting a 3-day "pooled" specimen of sputum for cytologic examination. Anyone who reported that he was unable to produce sputum spontaneously was offeredan for sputum induction<br>C: was not part of the surveillance group but advised to get the tests at trial entry.	Primary: incidence of lung cancer;<br>Secondary: Survival after treatment	Mortality from lung cancer
McNamara HC, 2015.	Norman JE, 2009.	Uncomplicated twin pregnancies recruited from specialized antenatal clinics for women with multiple pregnancy (from 9 hospitals)	I: intravaginal progesterone;<br>C: placebo	Primary: intrauterine death or delivery before 34 week<br>Maternal secondary outcomes: gestation at delivery, method of delivery (spontaneous vaginal delivery, vaginal	Primary: death, congenital anomalies and hospitalisation, routine national child health assessments.<br>Secondary: CDI and HUI

© 2018 Fitzpatrick T et al. *JAMA Network Open.*

Extension Study *	Original Trial*	Original Study Inclusion Criteria	Original Intervention	Original Trial Outcomes	Extended Trial Outcomes
				breech, forceps or ventouse, or caesarean section), duration of each stage of labour, and safety outcomes such as duration of stay in hospital, maternal satisfaction (by questionnaire).<br>Neonatal secondary outcomes: neonatal unit admission and duration of neonatal unit care.	
Miller AB, 2014.	Miller AB, 1992.	Women (50-59 years old) with no history of breast cancer and no mammography in the previous 12 months, recruited from 15 urban centers in Canada	I: annual mammography and physical examination (MP group);<br>C: annual physical examination only (PO group).	Rates of referral from screening, rates of detection of breast cancer from screening and from community care, nodal status, tumour size and rates of death from all causes and from breast cancer.	Deaths from breast cancer.
Myles PS, 2011.	Rigg JR, 2002.	Men and women (aged 18+) undergoing major abdominal surgery with one of nine defined comorbid states to identify high-risk status	I: intraoperative epidural anaesthesia and postoperative epidural analgesia for 72 h with general anaesthesia (site of epidural selected to provide optimum block);<br>C: Usual care	Death at 30 days or major postsurgical morbidity.	Cancer-free survival
Puntoni M, 2016.	Decensi A, 1994.	Men and women under 80 years of age, with non muscle invasive bladder cancer (stage Ta or T1, any grade) resected within the previous 3 years	i: fenretinide 200 mg/day<br>c: no treatment	Primary: flow cytometric DNA content in epithelial cells obtained after 12 months.<br>Secondary: recurrence	Primary: survival<br>Secondary: cancer survival, bladder cancer survival, prognostic value of circulating VEGF

© 2018 Fitzpatrick T et al. *JAMA Network Open.*

Extension Study *	Original Trial*	Original Study Inclusion Criteria	Original Intervention	Original Trial Outcomes	Extended Trial Outcomes
				rate, time to first positive cytology	
Sandblom G, 2011.	Pedersen KV, 1990.	Men aged 50-69 randomly selected from a population of 9026 in the area of Norrkoping.	I: digital rectal examination by a urologist and a general practitioner;<br>C: no examination	Primary: Prostates having a firm nodular consistency.<br>Secondary: costs, discomfort	Prostate cancer specific mortality, tumour stage, grade, and treatment.
Thomsen JL, 2005.	Lauritzen T, 1995.	Any Danish man or woman aged 30-50 years (as of Jan 1, 1991) registered with a general practitioner was included in the study.	i1: health check which included being screened for cardiovascular risk factors, lung and liver function, fitness, sight and hearing and an optional test for the human immunodeficiency virus (HIV); this group received written feedback from the general practitioner.<br>i2: also given a health check and written feedback; in addition, they were given the opportunity to attend their general practitioner to discuss preventive health.<br>c: not invited to participate	Primary: describe the baseline health status of participants including: presence of CVD risk factors, lung and liver function, fitness, sight, hearing and (optionally) their HIV status.<br>Secondary: report on the extent of health advice given as a result of a health check (and on how many healthy people did not require health advice following a health check) and on health goals that participants set following discussion of their health with their general practitioner.<br>The number of referrals to specialists following the health talks was also examined to determine whether the screening would result	Hospital admissions, Ambulatory contacts, Emergency room contacts

© 2018 Fitzpatrick T et al. *JAMA Network Open.*

Extension Study *	Original Trial*	Original Study Inclusion Criteria	Original Intervention	Original Trial Outcomes	Extended Trial Outcomes
				in an increase in the number of referrals.	
Virtamo J, 2014.	Virtamo J, 2003.	Male smokers aged 50-69 years living in 14 adjoining areas in south western Finland	I1: alpha-tocopherol (50 mg daily),<br>I2: Beta-carotene (20 mg daily);<br>I3: I1 and I2 combination<br>C: placebo daily	Primary: cancer<br>Secondary: overall and cause specific mortality	Cancer incidence and all cause mortality
**Therapeutic benefits lost in the trial extension phase**					
Clayton PA, 2012.	The Tricontinental Mycophenolate Mofetil Renal Transplantation Study Group, 1996.	Men and women receiving their first or second cadaveric renal transplant, were at least 18 years old, and were able to receive oral medication. Patients with a history of malignancy (except successfully treated nonmetastatic basal or squamous cell carcinoma of the skin), serologic evidence of human immunodeficiency virus or hepatitis B, systemic infections requiring continued antibiotic therapy at the time of entry, severe diarrhea, gastrointestinal disorders, or active peptic ulcer disease were excluded from entry, as were pregnant women, nursing mothers, and patients who did not agree to use adequate contraception.	i: Mycophenolate mofetil (MMF), an immunosuppressant, 1.5 g twice daily, MMF 1.0 g twice daily<br>c: azathioprine (AZA) 100-150 mg once daily	Primary: patient and graft survival; graft failure was defined as death or permanent loss of graft function.<br>Secondary: death-censored graft survival, cancer incidence, and estimated kidney function.	Patient survival, graft survival, non-skin cancer, skin cancer
Henderson RA, 2015.	Fox KA, 2002.	Men and women with suspected cardiac chest pain at rest and had documented evidence of coronary artery disease with at least one of: evidence of ischaemia on electrocardiograph (ST-segment	i: angiography within 48 h of index episode of cardiac chest pain<br>c: conservative management within 48 h	Primary: a combined rate of death, non-fatal myocardial infarction, or refractory angina at 4 months; and a combined rate of death	All-cause mortality, cardiovascular mortality

© 2018 Fitzpatrick T et al. *JAMA Network Open.*

Extension Study *	Original Trial*	Original Study Inclusion Criteria	Original Intervention	Original Trial Outcomes	Extended Trial Outcomes
		depression, transient ST elevation, left bundle branch block [documented previously], or T-wave inversion); pathological Q waves suggesting previous myocardial infarction; or arteriographically proven coronary artery disease on a previous arteriogram.	of the index episode of cardiac chest pain	or non- fatal myocardial infarction at 1 year. Secondary: angina, QOL, health-economic evaluations.	
Ishani A, 2006.	Hafström L, 1991.	Men and women with recurrent malignant melanoma of the extremities	I: Surgery plus regional perfusion<br>C: Surgery	Cancer progression and mortality	Mortality
McArdle CS, 2010.	McArdle CS, 1986.	Women admitted to teaching hospitals with operable breast cancer with axillary node involvement after surgery and no metastasis, aged 70 years or less	I1: post-op Radiotherapy,<br>I2: post-op chemotherapy<br>I3: both radiotherapy and chemotherapy after surgery for breast cancer	Disease free survival	Primary: cancer specific survival and all cause mortality.<br>Secondary: predictors of cancer mortality
Moss SM, 2015.	Moss S, 1999.	Women aged 39–41 years from 23 UK NHS Breast Screening Programme (NHSBSP) units (appendix) were identified from the general practitioners' (GP) lists of patients held in health authority databases.	i: invited for mammography screening<br>c: no invite for screening	Breast cancer mortality and breast cancer incidence (in situ, invasive, and total incidence).	Breast cancer mortality and breast cancer incidence
Roodnat JI, 2014.	Smak Gregoor PJ, 2002.	Men and women that underwent kidney transplantation in university hospitals (This is an open label study).	I1: withdraw cyclosporine from triple drug combo of cyclosporine, prednisone, and MMF<br>i2: withdrawal prednisone from triple drug combo<br>c: standard triple drug combo	Primary: first biopsy-proven acute or chronic rejection between randomization and 24 months after transplantation.<br>Secondary: patient and graft survival, renal function at 1 and 2 years after transplantation, the	Graft and patient survival.

© 2018 Fitzpatrick T et al. *JAMA Network Open.*

Extension Study *	Original Trial*	Original Study Inclusion Criteria	Original Intervention	Original Trial Outcomes	Extended Trial Outcomes
				incidence of infections and malignancies, and changes in BP and lipid metabolism.	
**Trial extension analyzed as observational study - i.e. not according to randomization**					
Bale G, 2008.	Burge PS, 2000.	Men and women aged between 40 and 75 years with mean forced expiratory volume in one second (FEV1) 50% of predicted normal	I: Fluticasone 500 ug BD; C: placebo	Primary: rate of decline in FEV1 after the bronchodilator and in health status, frequency of exacerbations, respiratory withdrawals. Secondary: Safety measures: morning serum cortisol concentration, incidence of adverse events.	All cause and cause specific mortality
Cygankiewicz I, 2009.	Moss AJ, 2002.	Men and women who were more than 21 years of age (there was no upper age limit) were eligible for the study if they had had a myocardial infarction one month or more before entry, as documented by the finding of an abnormal Q wave on electrocardiography, elevated cardiac-enzyme levels on laboratory testing during hospitalization for suspected myocardial infarction, a fixed defect on thallium scanning, or localized akinesis on ventriculography with evidence of obstructive coronary disease on angiography, and an ejection fraction of 0.30 or less within three months before entry, as assessed	i: implantable defibrillation c: conventional medical therapy	Death from any cause.	All-cause mortality

© 2018 Fitzpatrick T et al. *JAMA Network Open.*

Extension Study *	Original Trial*	Original Study Inclusion Criteria	Original Intervention	Original Trial Outcomes	Extended Trial Outcomes
		by angiography, radionuclide scanning, or echocardiography.			
Dalsgaard EM, 2014.	Griffin SJ, 2001.	General practices in the four study areas within a maximum of 100 miles of the study centres were invited to participate. All patients aged 40-69 years old with newly diagnosed with Type 2 diabetes in were eligible to participate, unless they are found to have: contraindications or intolerance to study medication; a history of alcohol- ism, drug abuse, psychosis or other emotional problems that are likely to invalidate informed consent or adherence to treatment; malignant disease with a poor prognosis; or are pregnant or lactating.	i: Intensive multifactorial diabetes treatment, including prescription of aspirin, lifestyle advice (concerning diet, physical activity and tobacco consumption) and stepwise increases in drug treatment of blood glucose, blood pressure and lipids according to strict targets.<br>c: conventional diabetes care - standard care according to broadly similar national recommendations for the management of Type 2 diabetes and prevention of cardiovascular disease.	Primary: All-cause mortality, Cardiovascular mortality/morbidity, Nonfatal myocardial infarction, nonfatal stroke, amputations, hospitalization for angina/congestive heart failure/coronary revascularization, peripheral revascularization.<br>Secondary: development of renal impairment, diabetic ulcers, blindness, reduced visual acuity, macular oedema, retinopathy, health status, health utility, quality of life, patient satisfaction, health service costs, patient costs	Change in cardiovascular risk factors (HbA1c, (mmol/mol (%)), cholesterol (mmol/l), blood pressure (mmHg), BMI and smoking status (current/former or never smoker)) from baseline to follow-up and the proportion of people meeting treatment targets; HbA1c ≤53 mmol/mol (7.0%), cholesterol ≤5.0 mmol/l and blood pressure ≤140/90mmHg.
Korpelainen R, 2010.	Korpelainen R, 2006.	A birth cohort of elderly women (born 1924-7, aged 70-73) who were residents of Oulu, Finland in Nov 1997 with hip BMD at least 2 SD below the reference value were included. Exclusion criteria: use of walking aid other than a stick, bilateral hip joint replacement, unstable chronic illness, medication	I: 30 months either of supervised and home-based impact exercise training<br>C: no intervention.	Primary: femoral neck, trochanter and total hip BMD.<br>Secondary: bone density measures at the radius and calcaneum	Fractures and death

© 2018 Fitzpatrick T et al. *JAMA Network Open.*

Extension Study *	Original Trial*	Original Study Inclusion Criteria	Original Intervention	Original Trial Outcomes	Extended Trial Outcomes
		known to affect bone density, severe cognitive impairment and involvement in other interventions.			
Kümler T, 2010.	Køber L, 1995.	Men and women with an enzyme confirmed acute myocardial infarction with wall motion index of less than or equal to 1.2, 6 days after MI.	i: trandolapril<br>c: placebo	All-cause mortality	All-cause mortality by wall motion index class, all-cause mortality by HF, survival by WMI, prognostic effect of WMI and HF
Nelson MR, 2015.	Wing LM, 2003.	Men and women aged 65+. Average systolic blood pressure of at least 160 mm Hg or an average diastolic blood pressure of at least 90 mm Hg (if the systolic blood pressure was at least 140 mm Hg); the absence of recent cardiovascular events (within the previous six months); and willingness to give informed consent.	i: ACE-inhibitor<br>c: diuretic agents<br>(The ACE inhibitor enalapril and the diuretic hydrochlorothiazide were recommended as initial therapy; however, the choice of the specific agent and dose was made by the family practitioner.)	Primary: all cardiovascular events and all-cause mortality.<br>Secondary: cause-specific cardiovascular events - initial and subsequent, fatal and nonfatal	Any first cardiovascular events such as stroke, myocardial infarction, heart failure, and any cardiovascular mortality and all-cause mortality
Novak M, 2013.	Wilhelmsen L, 1986.	All men living in Goteborg and born 1915-1922 and 1924-1925.	i: antihypertensive treatment in subjects with screening blood pressure above 175 mmHg systolic or 115 mmHg diastolic, dietary advice to men with serum cholesterol levels above 260 mg per 100 ml, advice to stop smoking to subjects who smoked more than 15 cigarettes per day.<br>C: no advice given	Incidence of non-fatal and fatal myocardial infarction, stroke and the total death rate in the intervention group compared with two control groups	Type 2 diabetes

© 2018 Fitzpatrick T et al. *JAMA Network Open.*

Extension Study *	Original Trial*	Original Study Inclusion Criteria	Original Intervention	Original Trial Outcomes	Extended Trial Outcomes
Regieli JJ, 2009.	Jukema JW, 1995.	All male patients who, from a review of the records at the participating centers, were scheduled to undergo coronary arteriography were considered for entry into the study; with serum cholesterol 4 - 8 mmol/l	I: Pravastatin 40 mg daily; C: placebo	Primary: 1) change in average mean segment diameter and 2) change in average minimum obstruction diameter; Secondary: Clinical events (MI, CHD death, PTCA or CABG, Stroke/ TIA; death)	Death from ischemic heart disease, all-cause mortality
Sharma A, 2012.	Menon U, 2009.	Post-menopausal women aged 50–74 years whose details were obtained from the registers of the participating 27 Primary Care Trusts. Women were excluded if they had a history of bilateral oophorectomy, active malignancy (women with a past history of malignancy were eligible if they had no documented persistent or recurrent disease and had not received treatment for >12 months), previous history of ovarian cancer, participation in other ovarian cancer screening trials, or increased risk of familial ovarian cancer. High-risk women were eligible for a separate trial: the United Kingdom Familial Ovarian Cancer Screening Study (UKFOCSS).	I1: annual CA125 screening (interpreted using a risk of ovarian cancer algorithm) with transvaginal ultrasound scan as a second-line test (multimodal screening [MMS]); I2: annual screening with transvaginal ultrasound (USS); C: usual care	Ovarian cancer mortality.	Primary epithelialovarian cancer (EOC) and slow growing borderline or Type I and aggressive Type II EOC
Smelov V, 2015.	Elfgren K, 2005.	Females 32 to 38 years, sexually active, who were recruited for screening in 5 major regions in Sweden; women with PAP smear taken in past 18 months were ineligible	I: Screening for HPV persistence and typing C: HPV test taken but not performed (sample frozen)	Intraepithelial neoplasia grade 2 or 3	CIN 2 and CIN3 risk according to HPV type

© 2018 Fitzpatrick T et al. *JAMA Network Open.*

Extension Study *	Original Trial*	Original Study Inclusion Criteria	Original Intervention	Original Trial Outcomes	Extended Trial Outcomes
White MC, 2005.	White MC, 2002.	Male and female jail inmates with latent TB infection	i: education every 2 weeks in jail.<br>co-i: incentive to be provided at the first visit to a TB clinic<br>c: no intervention	Primary: first visit to TB clinic after 1 month from prison release<br>Secondary: completion of a first course of therapy	Primary: rate of completed therapy.<br>Secondary: incidence of TB

† Individual extension studies may have reported outcomes belonging to multiple categories so the numbers in this table differ from those in Table 3 (Summary of results of trial extension studies), which categorizes all reported analyses (n=155). In this table studies were placed in categories according to a hierarchy in which superiority in the trial extension phase> harms in the trial extension phase> null effects in the trial extension phase> benefits lost in the trial extension phase > analyzed as an observational study. Thus, each study only appears once in the table (n=113).* References listed alphabetically by first author surname. For full references see Supplementary Table 3

© 2018 Fitzpatrick T et al. *JAMA Network Open.*
